# Balancing acts of SRI and an auto-inhibitory domain specify Set2 function at transcribed chromatin

**DOI:** 10.1093/nar/gkv393

**Published:** 2015-04-29

**Authors:** Yi Wang, Yanling Niu, Bing Li

**Affiliations:** 1 Biological Chemistry Graduate Program, UT Southwestern Medical Center, Dallas, TX 75390, USA; 2 Department of Molecular Biology, UT Southwestern Medical Center, 5323 Harry Hines Boulevard, Dallas, TX 75390, USA

## Abstract

Set2-mediated H3K36 methylation ubiquitously functions in coding regions in all eukaryotes. It has been linked to the regulation of acetylation states, histone exchange, alternative splicing, DNA repair and recombination. Set2 is recruited to transcribed chromatin through its SRI domain's direct association with phosphorylated Pol II. However, regulatory mechanisms for histone modifying enzymes like Set2 that travel with elongating Pol II remain largely unknown beyond their initial recruitment events. Here, by fusing Set2 to RNA Pol II, we found that the SRI domain can also recognize linker DNA of chromatin, thereby controlling Set2 substrate specificity. We also discovered that an auto-inhibitory domain (AID) of Set2 primarily restricts Set2 activity to transcribed chromatin and fine-tunes several functions of SRI. Finally, we demonstrated that AID mutations caused hyperactive Set2 *in vivo* and displayed a synthetic interaction with the histone chaperone FACT. Our data suggest that Set2 is intrinsically regulated through multiple mechanisms and emphasize the importance of a precise temporal control of H3K36 methylation during the dynamic transcription elongation process.

## INTRODUCTION

Histones are subjected to various post-translational modifications (PTMs) such as methylation, acetylation and phosphorylation. It was proposed that individual histone modifications or combinations of modifications mainly serve as a signaling platform for the regulation of chromatin structure and almost all DNA-related processes (1). Methylation of lysine 36 on histone H3 (H3K36me) is an important histone ‘mark’ that is ubiquitously present in all eukaryotes. In yeast, Set2 is responsible for all three states of methylation at H3K36 (mono-, di- and tri-methylation) (2). H3K36 methylation is transcription-coupled and enriched at the middle and 3′ end of RNA Pol II genes that are actively transcribed (3). One of the key functions of H3K36me is to recruit and activate a histone deacetylase complex, Rpd3S, to deacetylate transcribed chromatin in the wake of Pol II passage (reviewed in (4)). The resulting hypo-acetylated states at transcribed regions prevent spurious transcription from cryptic promoters (5–8). Recently, H3K36me was also shown to suppress the exchange of newly synthesized histones by decreasing the binding of H3 to histone chaperone Asf1 and recruiting the Isw1 chromatin remodeler (9,10). In higher eukaryotes, multiple SET domain-containing methyltransferases that methylate H3K36 at different states have been identified (11). H3K36 methylation that is mediated by Set2 homologs has been shown to regulate dosage compensation (12,13), alternative splicing (14) and the activities of the PRC2 H3K27 histone methyltransferase complex (4). Recently, H3K36me was also linked to DNA damage repair and maintenance of genome stability (15–17).

H3K36 methylation is tightly regulated through multiple mechanisms. Set2 directly associates with elongating Pol II through its Set2-Rpb1 interaction domain (SRI), which binds to the phosphorylated C-terminal domain of the largest subunit, Rpb1 (CTD) (18–21). This association is crucial for the full activity and stability of Set2 (22). Disruption of CTD kinases, such as CTDK-I and the Bur1 complex, causes defects in methylation of K36 to K36me2 and K36me3 (23–25). Similarly, mutation of the Paf1 complex, which regulates the phosphorylation status of CTD, eliminates H3K36me3 but not K36me2 (24,26). Interestingly, ectopic expression of the SET domain alone can recover H3K36me2 to the wild-type level at transcribed regions and with a normal distribution pattern (23), suggesting that only H3K36me3 requires Set2 to associate with phosphorylated CTD (p-CTD). A recent study provided a plausible explanation for this methyl-state-dependent requirement. Fuchs *et al*. showed that Set2 has a short half-life, which is controlled by a proteasome-dependent pathway through the SRI domain (22). It was proposed that free Set2 (T_1/2_ ∼ 9 min) is capable of di-methylating H3K36. However, the binding of Set2 to the hyper-phosphorylated Pol II not only stabilizes the Set2 protein but also increases its resident time at transcribed regions, which appears to be essential for the formation and maintenance of H3K36me3 (22). Lastly, Set2 also needs to make multiple contacts with nucleosomal substrates to achieve its full activity. Mutations of Set2 histone interaction motifs or the histone surface residues that contact Set2 lead to the decreased levels of the various methylation states of H3K36 (27–29). Similarly, H3K36me3 is also influenced by the mutation of histone chaperones that modulate nucleosome assembly/disassembly, such as Asf1 (30) and FACT (25)

All Set2 homologs contain a highly conserved catalytic SET domain, WW domain, coiled-coil domain, SRI and a large middle region of unknown function. A recent study reports that the proline-rich motif within the middle region of human Setd2 inhibits the binding of the WW domain to Huntingtin (Htt) protein (31). The middle region of the Set1 histone methyltransferase was shown to serve as an auto-inhibitory domain (AID) that represses trimethylation of H3K4 (32,33). In this study, by fusing Set2 to RNA Pol II, we found that the SRI domain, beside its known role in binding to Pol II, is also responsible for recognizing linker DNA of chromatin and controlling Set2 substrate specificity. We also discovered that the middle domain of Set2 primarily functions as an AID to restrict Set2 activity to transcribed chromatin. Mechanistic studies revealed that AID mediated the repression by antagonizing the catalytic activity of SET and fine-tuning several functions of SRI. Interestingly, we found that disruption of different portions of AID all resulted in hyper-active Set2 *in vitro*. However, these mutations caused distinct patterns of CTD binding and protein stability *in vivo*. Finally, we showed that AID mutants display synthetic genetic interactions with histone chaperone FACT, underscoring the importance of precise temporal control of K36 methylation during the dynamic elongation process.

## MATERIALS AND METHODS

Plasmids and yeast strains were constructed based on standard procedures and are listed in Supplementary Tables S1 and S2, respectively. Protein purification, ChIP, CTD peptide pull-down and other standard procedures for nucleosome preparation and Electrophoretic mobility shift assay (EMSA) are described in the Supplementary Data.

### Histone methyltransferase assays (HMT)

Standard histone methyltransferase assays (HMT) reactions were carried out in a 20 μl system using 1× HMT buffer (50 mM Tris–HCl pH 8.0, 50 mM NaCl, 1 mM MgCl_2_, 2 mM Dithiothreitol (DTT) and 5% glycerol) supplemented with either 0.5 μl of radio-labeled ^3^H S-Adenosyl methionine (80 Ci/mmol, Perkin Elmer) or 10 μM cold S-Adenosyl methionine (Sigma) (20). A scintillation counting-based filter-binding assay was performed as described previously (34). To detect specific methylation states on given histone substrates, western blots were performed using antibodies against H3K36me3 (Abcam 9050) and H3K36Me2 (Abcam 9049). For the Tobacco Etch Virus Protease (TEV)-digestion HMT experiments described in Figure 4, WT Set2 and its derivatives that each contains an internal TEV cleavage site were incubated with 2.5 units of TEV protease (Life Technologies) in 10 μl of TEV Buffer (10 mM Tris–HCl pH8.0, 0.1% NP40 and 1 mM DTT) at 30°C for 1 h. Half of the reactions were directly mixed with 3× sodium dodecyl sulphate loading buffer for sodium dodecyl sulphate-polyacrylamide gel electrophoresis. For the other half of each reaction, 15 μl of HMT master mix (for each reaction: 4 μl of 5× HMT Buffer, 0.5 μl of ^3^H S-Adenosyl methionine, 1 μg of HeLa oligonucleosomes and 9.8 μl H_2_O) was then added and incubated at 30°C for 1 h. ^3^H-incorporation was measured via filter-binding assays. For salt-titration experiments as shown in Figure 2A, 5× HMT-0-NaCl buffer (250 mM Tris–HCl pH 8.0, 5 mM MgCl_2_, 10 mM DTT and 25% glycerol) was used, and NaCl concentration from each substrates (HeLa nucleosomes 0.6 M NaCl; HeLa core histone 2.5 M, recombinant nucleosomes no salt and recombinant histone octamers/tetramers 2 M) was taken into the consideration of the final salt condition, was adjusted by adding 5 M NaCl.

### Chromatin immunoprecipitation (ChIP)

Two hundred milliliters of each yeast culture were grown to reach OD_600_ = 1.0. 1% formaldehyde was used for crosslinking at RT for 15 min. Standard ChIP procedures were described previously (7). A total of 1 μl of anti-H3 (Abcam 1709), 0.5 μl of anti-trimethylated H3K36 (Abcam 9050), 1.5 μl of anti-dimethylated H3K36 (Abcam 9049) were used for immunoprecipitation. Input DNA and immunoprecipitated DNA were quantified using multiplex PCR. Primer mixtures containing 5 primer pairs corresponded to: −879 and −745 of the *STE11* promoter (A)(P1397/1398), 87 and 330 of *STE11* 5′ (B)(P1399/1400), 925 and 1111 of *STE11* coding region (C)(P1401/1402), 1839 and 2129 of *STE11* 3′ (D)(P1403/1404), 4193 and 4477 of the ORF-free region of ChrI (Z) (P1740/1741). Band intensity was quantified using the UN-SCAN-IT V6.1software (Silk Scientific, Inc.)

## RESULTS

### Pol II-Set2 contact regulates Set2-mediated H3K36me3 beyond the initial recruitment and stabilization of the protein

**Figure 1.**
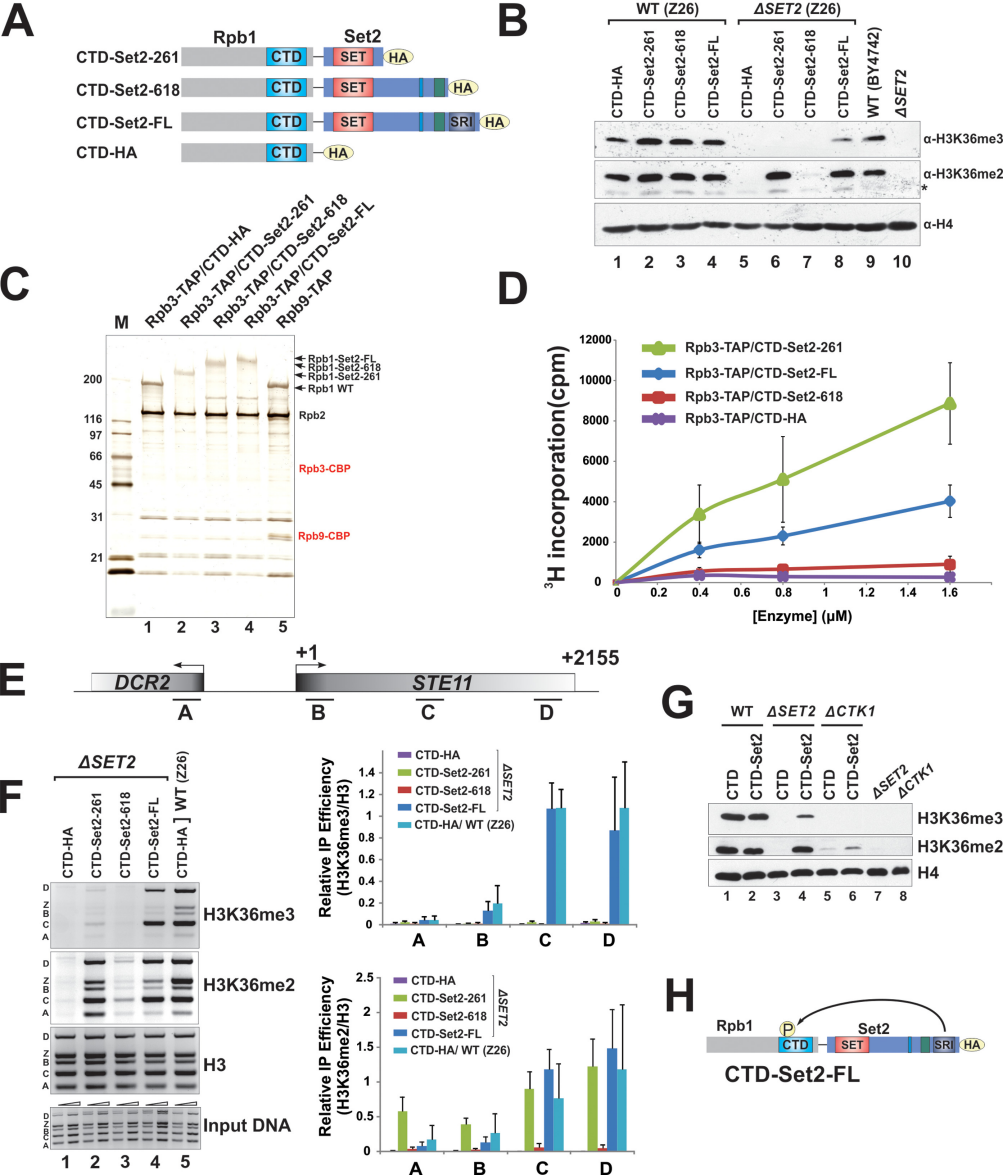
The binding of SRI to phosphorylated CTD plays additional roles in Set2-mediated H3K36me3 beside stabilization and recruitment of Set2. (**A**) A scheme for artificial recruitment of Set2 to Pol II by fusing the indicated Set2 constructs to the C-terminus of Rpb1, the largest subunit of RNA polymerase II. (**B**) Fusing the catalytic domain of Set2 to Pol II is not sufficient for H3K36me3. Plasmids including pWY043 (pYIA CTD-*SET2* FL), pWY044 (pYIA CTD-*SET2* 1–618), pWY045 (pYIA CTD-*SET2* 1–261) and pWY046 (pYIA CTD) were transformed into wild-type (YYW120, Z26) and *ΔSET2* (YYW121). Whole cell extracts were subjected to western blot using the indicated antibodies. (**C**) Silver staining of TAP-purified Pol II complexes. The indicated Pol II-Set2 fusion plasmids were introduced into the yeast YYW122 (a Pol II shuffle strain), which contains Rpb3-TAP for purification. (**D**) HMT assays using the PolII-Set2 fusion complexes as shown in (C) and HeLa oligonucleosome substrates. (**E** and **F**) H3K36 methylation status in Pol II-Set2 fusion strains was monitored by ChIP assays; (E) Illustration of PCR-amplicons around the *STE11* region (label as ‘A’ to ‘D’) used in ChIP assays to monitor K36me; (F) Multiplex-PCR with the indicated primer sets were performed to quantify the amount of immunoprecipitated DNA. The Z region (labeled as ‘Z’) located at a gene desert served as an internal control. However, we consistently detected H3K36me at this locus, which is likely due to uncharacterized non-coding RNA transcription events. The relative IP efficiency is the ratio of IP efficiency of given antibodies at specific loci over the IP efficiency of histone H3. Data are represented as mean ± SEM with at least three independent experiments. (**G**) CTD phosphorylation mediated by CTDK-I is required for H3K36me3 even when full-length Set2 is artificially fused to Pol II. pWY046 (vector control) and pWY043 (CTD-Set2) were transformed into Δ*CTK1* and control strains. Western blots of whole cell extracts of indicated strains. (**H**) A proposed model showing how Pol II-fused Set2 interacts with Rpb1 through phosphorylated CTD.

It was proposed that the binding of Set2 to phosphorylated Pol II stabilizes Set2 protein and increases its resident time at transcribed regions (22). An interesting study showed that fusing an mRNA capping enzyme to the C-terminus of CTD can bypass the lethality caused by the CTD S5A mutation, which disrupts the recruitment of the capping enzyme to elongating Pol II (35). We therefore wondered if the artificial recruitment of Set2 to Pol II CTD could totally bypass the requirement for SRI function and CTD phosphorylation. To this end, we designed a series of plasmid constructs in which three versions of Set2 (full length (CTD-FL), SET domain alone (CTD-SET) and deletion of the SRI region (CTD-Set2–618), which should abolish Set2-Pol II contacts) were fused to the C-terminus of Rpb1 (Figure 1A). It has previously been shown that Set2–618 (1–618aa) has no activity *in vivo* (18), presumably due to the enzyme failing to be recruited to the elongating Pol II. Hence, we rationalized that fusing it to Pol II might be able to circumvent the need of SRI for Pol II binding. These plasmids were transferred into an Rpb1-shuffle strain in which *SET2* was deleted, and the Set2 fusion was the only H3K36 methylase in each resulting cell. Analysis of the bulk levels of H3K36me revealed that the CTD-SET fusion only recovered K36me2 but not K36me3 (Figure 1B, Lane 6 and S1A), which resembled what was observed when the SET domain alone was expressed in free form (23). However, when we fused Set2–618 to Rpb1, surprisingly, no methylation of K36me was observed (Figure 1B, Lane 7), which indicated that recruiting to Pol II is not sufficient for Set2 activity and SRI may play additional roles in regulating Set2 activity. As a control, we found that fusing full length Set2 to Pol II led to almost wild type levels of K36me2 and a slightly lower level of K36me3 (Figure 1B, Lane 8).

To rule out the possibility that fusing Set2 to CTD may disrupt the proper folding of the catalytic SET domain, we purified all three Pol II fusions using TAP-tagged Rpb3. Silver staining showed that there was no detectable endogenous Set2 that was co-purified with the largely unphosphorylated Pol II (Figure 1C), which is consistent with a previous report (20). Fusing Set2 to CTD did not appear to alter Pol II stability because the stoichiometries of the Pol II fusions (from looking at Rpb1 and Rpb2 levels) were comparable to that of wild-type Pol II (Figure 1C, Lane 5). The HMT activities of these CTD-fused Set2 were subsequently measured. Consistent with the *in vivo* methylation level observed above, CTD-Set2–618 only showed a background level of HMT activity, whereas both CTD-SET and -FL fusions displayed strong activities (Figure 1D). We noticed that CTD-SET appeared to be more active than the FL fusion protein. We speculated that in the CTD-FL fusion, the SET domain is situated in the middle of the fusion protein. This configuration may likely restrict its mobility, which is often important for catalysis.

**Figure 2.**
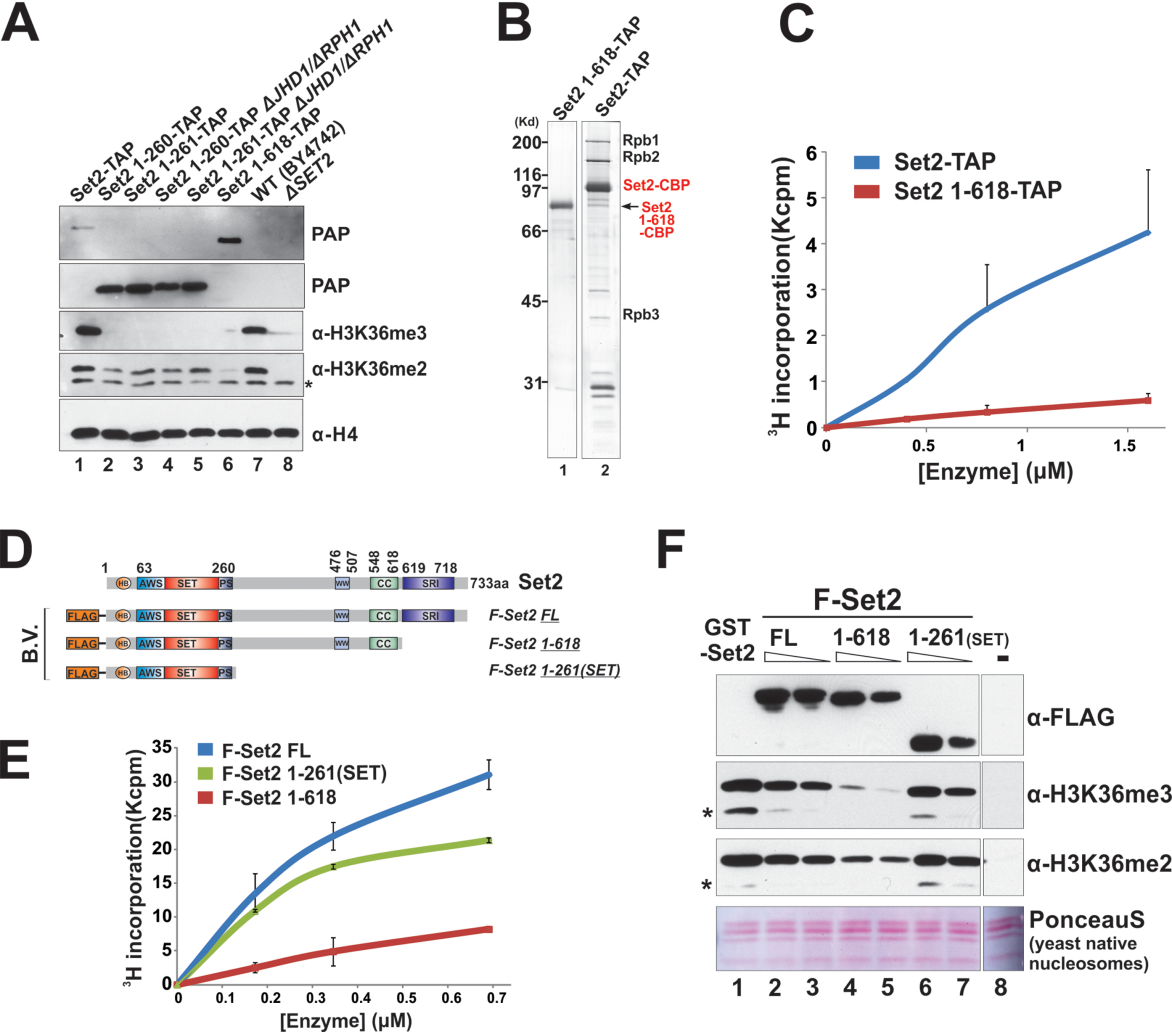
Set2 contains an auto-inhibitory domain for the H3K36 methyltransferase activity. (**A**) The Set2 ΔSRI mutant shows severely compromised H3K36 methylation *invivo*. Whole cell extracts from indicated yeast strains were subjected to western blotting analyses. (**B**) Silver staining of TAP-purified Set2 WT and ΔSRI complexes. (**C**) HMT assays using HeLa oligonucleosome substrates. HMT activity is represented as mean ± SEM, *n* = 3. (**D**) An illustration of recombinant Set2 and truncated derivatives. The SET domain, histone binding motif (HB), AWS domain, post SET domain (PS), WW domain, coiled-coil motif (CC) and Set2-Rpb1 interaction domain (SRI) are shown along with their positions within full-length Set2. (**E**) Standard HMT assay using HeLa oligonucleosome substrates. (**F**) HMT assay using native yeast nucleosomes that were extracted from *ΔSET2* so that they do not carry any H3K36me. The reaction products were detected using western blotting with the indicated antibodies. Ponceau S-stained yeast nucleosomes in each reaction served as loading controls. Lane 8 was from the same gel and the intervening lanes were omitted which contain irrelevant controls.

We next sought to test if the H3K36 methylation status at individual genomic loci follows a similar trend as the bulk levels that we measured. ChIP was performed to monitor the level of K36me at a model gene, *STE11*. As shown in Figure 1E and F, the methylation patterns at *STE11* in all three mutants were generally consistent with bulk methylation levels. Similar to wild-type cells, CTD-Set2-FL resulted in enrichment of H3K36me2 and H3K36me3 at coding regions that peaked toward the 3′ ends (36). K36me2 and K36me3 were nearly abolished in CTD-Set2–618 cells. Fusing SET to CTD only resulted in the presence of K36me2 but no K36me3 (Figure 1F), suggesting that SET alone is not sufficient for any H3K36me3 even when it was tethered by Pol II and stayed at transcribed region with presumably longer resident time. To further confirm this, we showed that expression of SET alone could not restore the levels of H3K36me3 even in the absence of the histone demethylases Rph1 and Jhd1 at the normal SET expression level (Figure 2A) or when it was overexpressed (Supplementary Figure S1B). Interestingly, we noted that CTD-SET caused elevated levels of K36me2 at promoter regions, whereas CTD-FL did not displayed this abnormal pattern (Figure 1F, A and B regions at *STE11*), suggesting that additional mechanisms within wild-type Set2 prevent such an aberrant activity.

To evaluate if phosphorylation of CTD by CDTK-I was still required for Set2 activity when it was fused to Pol II, we transformed the CTD-Set2-FL plasmid into *ΔCTK1* cells. Similar to a previous report (22), the endogenous Set2 was degraded and no K36me could be detected in *ΔCTK1* alone (Figure 1G, Lane 8). Fusing Set2 to Pol II stabilized the Set2 protein (Supplementary Figure S1D), and modestly recovered the levels of K36me2, but no K36me3 was detected (Figure 1G, Lane 6), indicating that p-CTD/SRI engagement is essential for full activity of Set2 even if it is fused to Rpb1. In this case, such an interaction might be due to SRI looping back to its CTD target (Figure 1H). Another piece of information that supports this possibility comes from our study on the Paf1 complex. Deletion of Paf1 complex subunit Cdc7 or Paf1 has been shown to cause defects of CTD Ser2 phosphorylation and destabilization of Set2 (22). We demonstrated that overexpression of Set2 restored the levels of Set2 in Δ*CDC73* or Δ*PAF1* but failed to recover K36me3 in the absence phosphorylation of serine 2 of the CTD (Supplementary Figure S1C). Collectively, these results strongly suggested that Set2/p-CTD contact beyond just the recruitment of Set2 to Pol II is necessary for K36me3.

### Set2 contains an auto-inhibitory domain for the H3K36 methyltransferase activity

We showed that fusing Set2–618 to CTD gave rise to minimal K36me2 and K36me3 recovery (Figure 1B). One possibility is that Set2–618 is not an active enzyme, which is consistent with a previous *in vivo* observation that Set2–618 caused a total loss of K36me2 and K36me3 even when it can be ChIP to coding region (22,23). We speculated that the middle portion (262–618 aa) of Set2 may internally repress the SET domain activity. To test this hypothesis, we took two independent approaches to purify Set2–618 and test its HMT activity. First, we constructed a yeast strain in which Set2–618 was TAP-tagged. Western blots analysis confirmed the previous findings that no K36me2 and K36me3 were detected in this strain (Figure 2A). We then purified native Set2–618 and full-length control using TAP purification, and as expected, Set2–618 was not associated with Pol II (Figure 2B, Lane 1). A subsequent HMT assay revealed that, compared to full-length Set2-TAP, Set2–618-TAP displayed minimal activity on HeLa nucleosomes (Figure 2C), suggesting that Set2–618 is repressed. To avoid the complication introduced by the presence of p-Pol II that was co-purified with full-length Set2, we next sought to purify the recombinant Set2 forms through two independent approaches. We first prepared all Set2 derivatives using a baculovirus expression system in insect cell culture (Figure 2D), which yielded highly purified Set2 with minimal degradation products, particular for Set2–618 (Supplementary Figure S2B, Lane 2). Using these recombinant forms and HeLa oligonucleosomes as substrates, we demonstrated that Set2–618 was much less active than FL Set2 and the SET domain alone (Figure 2E). To test these enzymes with more physiologically relevant substrates, we prepared yeast native nucleosomes from a *ΔSET2* yeast strain and demonstrated that no K36me was detected from the resulting nucleosomes (Figure 2F, Lane 8). Using these native yeast nucleosomes, we showed that Set2–618 also displayed much weaker activity comparing to SET and Set2-FL, particularly for K36me3 (Figure 2F). We also purified bacterially expressed GST-Set2–618, and showed the similar result in HMT assays (Supplementary Figure S2C). In conclusion, we demonstrated that the enzymatic activity of Set2–618 is repressed, and its activity is controlled by the middle region (262–618 aa).

### Full-length Set2 and Set2 SET domain display distinct substrate specificities

**Figure 3.**
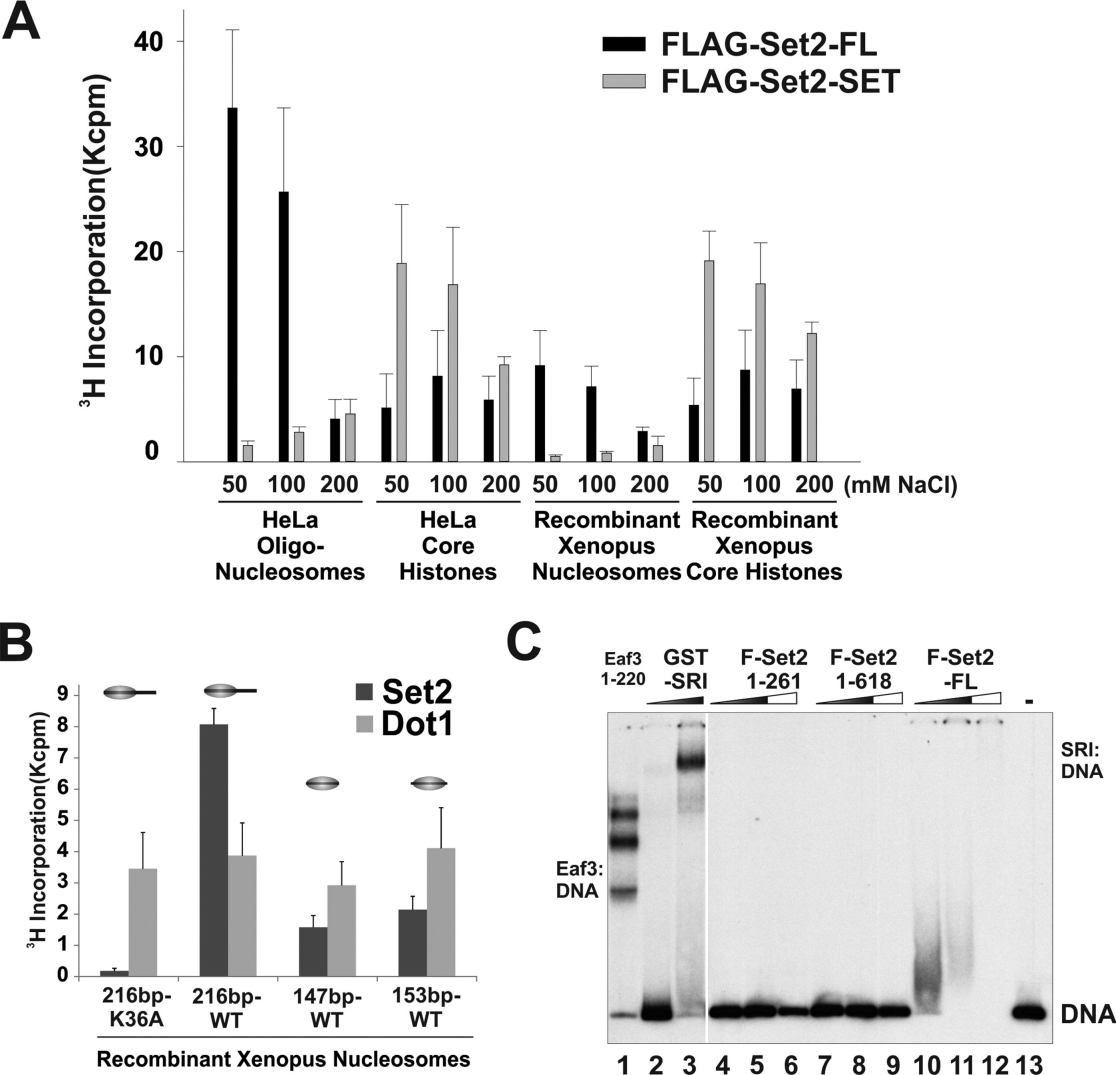
SRI DNA binding ability confers the preference of Set2 to nucleosomal substrates. (**A**) Under low salt conditions, full-length Set2 prefers nucleosomes, but the Set2 SET domain shows more activity toward core histones. HMT assays were carried out under the indicated salt concentration with equal amounts of each nucleosome/histone substrate. (**B**) Nucleosomal activity of Set2 depends on the length of linker DNA. Recombinant mono-nucleosomes with the indicated DNA templates were used in HMT reactions. Recombinant GST-Dot1 was used as a control. (**C**) EMSA assay showing that SRI is responsible for the binding of Set2 to DNA. End-labeled 216-bp DNA that contains a 601 positioning sequence was used. The truncated Eaf3 proteins served as positive controls for DNA binding.

While testing Set2 activity on different histones and nucleosomal substrates, we noticed that like many other histone modifying enzymes, Set2 activity was very sensitive to salt concentration (Figure 3A). Curiously, Set2-FL showed decreased activity when salt concentration increased; whereas the SET domain gained more activity under higher-salt conditions (Figure 3A). Importantly, at all of the conditions that we tested, particularly at the low-salt concentration (50 mM), Set2-FL displayed a strong preference for nucleosomal substrates over core histones while the SET domain showed the opposite pattern, favoring core histone substrates. Consistently, SET was also more active on recombinant H3/H4 tetramers than Set2-FL (Supplementary Figure S2D). We postulated that the linker DNA might contribute to the substrate preference of Set2. Thus, we reconstituted four different kinds of recombinant nucleosomes with varying linker lengths. As shown in Figure 3B, Set2-FL was more active on nucleosomes that have longer linker DNA, while another histone methyltransferase, Dot1, worked equally well on all nucleosomes tested. To find out which part of Set2 is responsible for DNA recognition, we performed gel-mobility shift experiments using radio-labeled DNA. Set2-FL bound to DNA strongly, while Set2-SET and Set2–618 showed weak binding as judged by the reduced intensity of unbound DNA in Lanes 6 and 9, respectively (Figure 3C). Interestingly, the CTD binding domain SRI also binds to DNA strongly (Figure 3C), which is consistent with the predicted DNA binding ability of this region (Supplementary Figure S2E). This result provided one plausible reason why Set2-FL prefers the nucleosomal substrates with longer linker DNA.

### The middle region of Set2 intrinsically suppresses the activity of the SET domain

**Figure 4.**
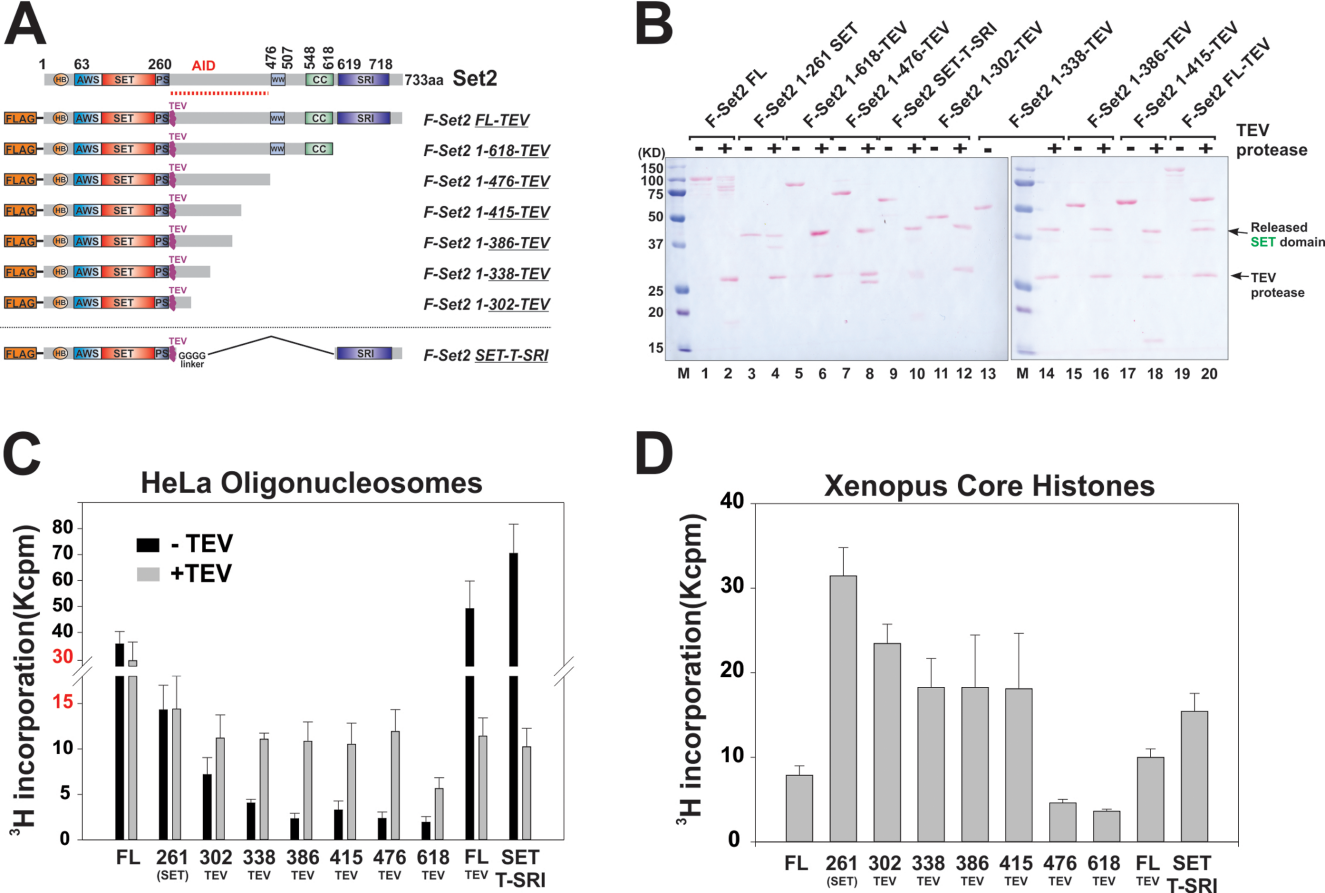
The middle region of Set2 intrinsically suppresses the activity of the SET domain. (**A**) A schematic illustration of Set2 and mutants in which a recognition site for TEV protease is engineered right after the SET domain. (**B**) Ponceau S staining showing the completion of TEV digestion. The bands representing TEV-cleaved SET domain and TEV proteases are labeled. (**C**) Auto-inhibition domain of Set2 represses the SET activity in cis. HMT assays using HeLa nucleosome substrates to monitor the effect of TEV cleavage on Set2 activity. Data are represented as mean ± SEM. (**D**) HMT assay with indicated Set2 derivatives and recombinant *Xenopus* core histone substrates.

Since SET alone is active while Set2–618 is repressed (Figure 2), we wondered if the SET region within Set2–618 was mis-folded during purification, which might in turn cause reduced activity. To address this possibility, we developed a strategy in which a TEV cleavage site was inserted between SET and the middle region. If the SET region within Set2–618 is properly folded and active but suppressed by the middle region, we would expect to see increased activity when TEV protease is used to cleave off the middle region (Supplementary Figure S3A). In the meantime, we were also interested in pinpointing the specific regions that are responsible for this potential repression. Based on secondary structure prediction, we divided the middle region into five hypothetical alpha-helix motifs (named motifs A–E; Supplementary Figure S2E) and created a series of C-terminal truncations according to these five motifs as illustrated in Figure 4A. All mutant Set2 were purified to homogeneity (Supplementary Figure S3B) and incubated with TEV digestion buffer in the absence or present of the TEV protease. As shown in Figure 4B, completion of TEV digestion was confirmed by Ponceau S staining and all of the SET fragments were released from the original middle-region truncations. HMT assays were then performed first using nucleosomal substrates. Without TEV digestion (Figure 4C, black bars), Set2–618 TEV was in a repressed state compared to SET (261) alone, which was consistent with our observations above (Figure 2). It appeared that this repression was progressively reduced as the middle region was trimmed smaller. More importantly, when TEV was applied, most truncations resumed their activity to nearly the level of SET domain alone (Figure 4C, gray bars), indicating that the cleaved middle regions intrinsically repressed SET activity. When core histone substrates were used in HMT assays, a similar trend of repression was observed among the mutants (Figure 4D). We noticed that full-repression on histones appeared to require a larger part of the middle region (262–476 aa) than that for nucleosomal substrates (262–386 aa). Since the SET domain prefers histone substrates, we therefore defined the region from 262–476 aa as the AID (Figure 4A). Given that the spatial location of H3K36 within the nucleosome structure makes it difficult to access, it is conceivable that it may be more difficult for AID to block SET activity toward H3K36 in histones than H3K36 within nucleosomes.

We also prepared a mutant in which the entire middle region was deleted, and the SET and SRI regions were joined together by a poly-glycine linker and a TEV site (SET-T-SRI). Remarkably, we found that SRI in this construct strongly stimulated SET activity on nucleosomes (Figure 4C) but inhibited its activity toward histone substrates (Figure 4D). This stimulatory effect of SRI can be explained by its affinity to linker DNA. However, the surprising SRI-mediated repression on core histones revealed a new mechanism for controlling Set2 substrate specificity. Since Set2-FL was fully active on nucleosomes, these data also indicated that the SRI domain can directly antagonize AID functions.

### AID regulates Set2 activities through multiple mechanisms

**Figure 5.**
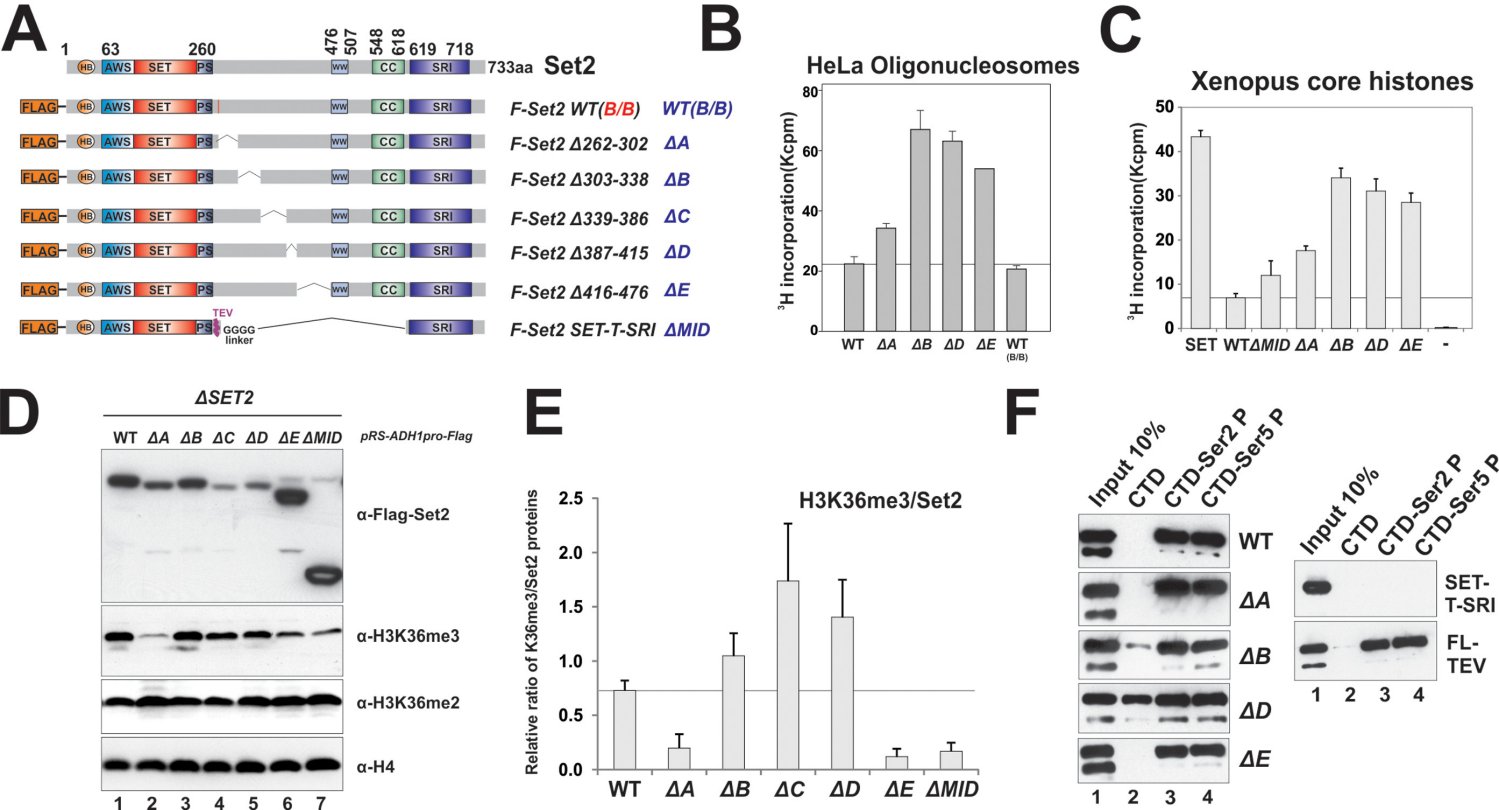
AID plays multiple roles in regulating Set2 activity *invitro* and *invivo*. (**A**) Set2 constructs for mapping minimal deletion that causes de-repression of SET activity. (**B** and **C**) Integrity of the AID domain is essential for regulating Set2 activity in vitro as measured by HMT assays using different substrates: (**B**) HeLa oligonucleosome substrates; (**C**) recombinant *Xenopus* core histones. (**D** and **E**) Set2 AID mutants gave rise to distinct H3K36 methylation pattern *invivo*: (D) western blot showing the levels of tagged Set2 proteins and H3K36me levels in the mutants; (E) Quantification of H3K36me3 results shown in (D) based on at least four independent experiments. Data are represented as mean ± SEM. (**F**) AID regulates the binding of Set2 to Pol II. Peptide pull-down experiments were performed using CTD peptides that consist of four heptad repeats, which are unmodified, phosphorylated at Ser2 (Ser2P) or phosphorylated at Ser5 (Ser5P).

We have mapped AID to the 262–476 aa region of Set2 (Figure 4). To create AID mutants that were more conducive for *in vivo* studies, we decided to further analyze the AID regions and identify the minimal deletion that can compromise AID functions. To this end, we constructed a series of internal mutations in which each individual hypothetical alpha-helix motif (A–E) or combinations were deleted (Figure 5A). All mutants were expressed in insect cells and purified to homogeneity (Supplementary Figure S3C and D), except for the mutant ΔC, which was always severely degraded despite multiple attempts. We performed the HMT assay using nucleosomal substrates and found that single deletion of B or D motifs de-repressed the enzyme to a level that almost resembled total deletion of AID (ΔAE) (Figure 5B and Supplementary Figure S3E); ΔA and ΔE also de-repressed the enzyme but to a lesser extent (Figure 5B). These results indicated that all helical motifs contributed to the overall repression, but B and D motifs might be crucial to coordinate the proper functions of other regions. Interestingly, all deletion constructs showed elevated HMT activity on core histones (Figure 5C), suggesting that AID mutations promote aberrant methylation of free histones by Set2.

We next introduced these AID mutants into a Δ*SET2* yeast strain for *in vivo* analysis. These mutants were initially expressed under the control of the native *SET2* promoter, and all mutants showed decreased stability (Supplementary Figure S4A–C). We then switched to a strong constitutive *ADH1* promoter to drive higher expression levels, which led to stable accumulation of most mutants to near wild-type level, except for ΔC and ΔD (Figure 5D, top panel). These two lines of evidence revealed that AID also plays an important role in regulating Set2 stabilization *in vivo*. Because most of these mutants showed great stability in a baculovirus system and were highly active *in vitro*, we believed that the instability of these mutants *in vivo* was unlikely due to mis-folding of the proteins (see more in the ‘Discussion’ section). When K36 methylation was measured, all mutants showed levels of K36me2 comparable to that in wild-type cells (Figure 5D). However, the pattern of K36me3 among those mutants was surprisingly varied. Considering that these mutants are all hyperactive *in vitro*, ΔB, ΔC and ΔD showed increased K36me3 levels after normalized to the level of Set2 (Figure 5E). On the other hand, ΔE and ΔMID could stably accumulate; however, both mutants showed lower levels of K36me3. To reconcile these distinct outcomes caused by different AID mutations, we considered that AID mutations may also influence the binding of mutant Set2 to Pol II. We therefore performed CTD peptide pull-down experiments using purified mutant proteins. We have shown previously that wild-type Set2 binds to Ser2- or Ser5-phosphorylated peptides but not to unphosphorylated CTD (Figure 5F) (20). ΔA and ΔE mutants behaved similar to wild-type. However, ΔB and ΔD non-specifically bound to unphosphorylated CTD (Figure 5F). To map the region that mediates unphosphorylated CTD binding, we further created a series of Set2 N-terminal truncations and demonstrated that AID (262–476 aa) was also responsible for contacting unphosphorylated CTD (Supplementary Figure S5). We showed earlier that SRI greatly stimulated SET activity on nucleosomes in the SET-SRI fusion (Figure 4C). Strikingly, this SRI-SET interaction appeared to completely block the binding of SRI to p-CTD (Figure 5F and Supplementary Figure S5C). This aberrant CTD binding pattern partially explained the low level of H3K36me3 in the ΔMID mutant. In summary, AID mutations generally resulted in hyperactive Set2 *in vivo*. However, K36me3 levels in each mutant were complicated by the changes of protein stability and CTD binding caused by AID mutations.

### Mis-regulation of H3K36me leads to synthetic phenotypes between AID mutants and a histone chaperone mutant

**Figure 6.**
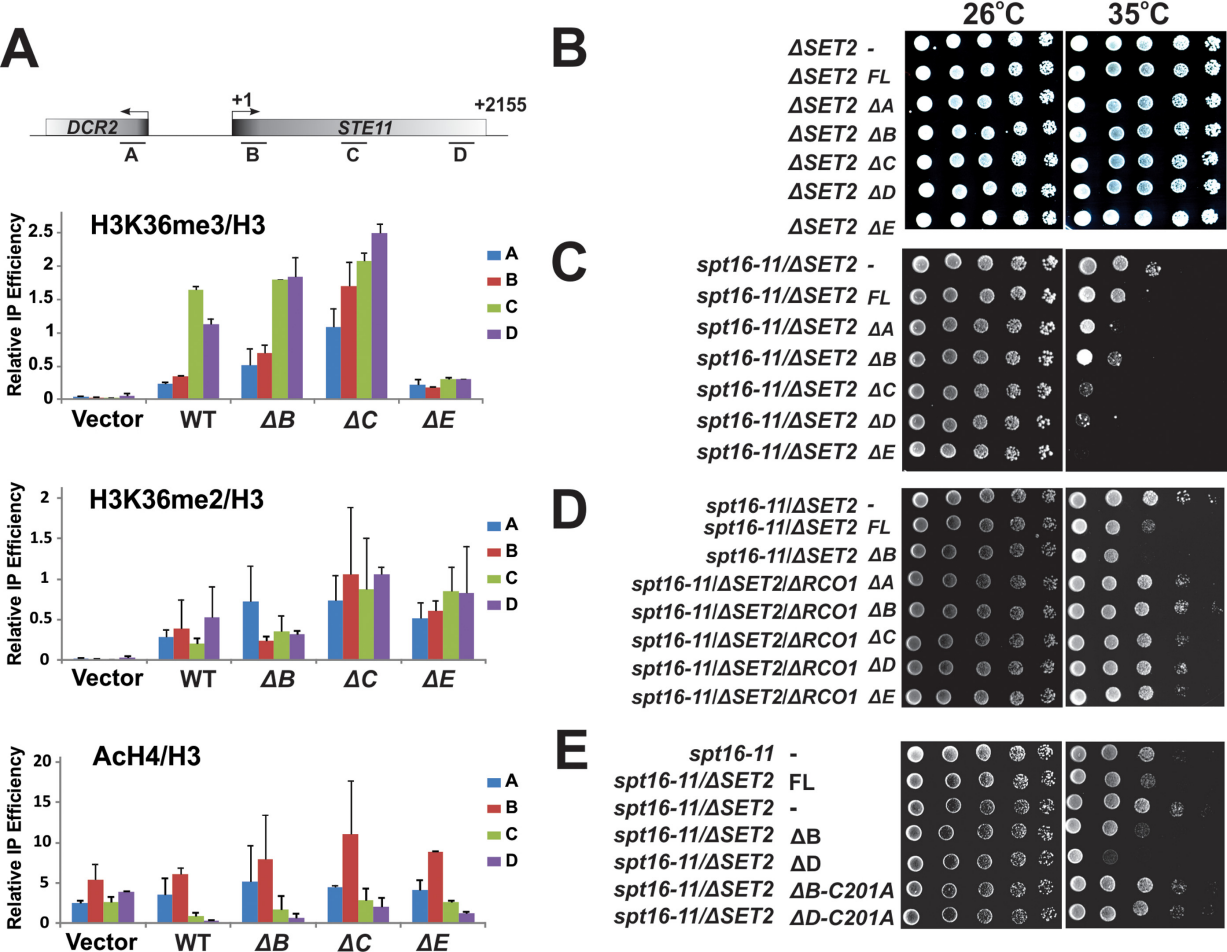
Aberrant H3K36 methylation caused by AID mutations leads to hyper-methylation phenotype. (**A**) Histone modification status at the *STE11* region in AID mutants was determined by ChIP assays. IP efficiencies were normalized to that of histone H3 and shown as mean ± SEM, *n* = 3. (**B**–**E**) AID mutants showed synthetic phenotypes with histone chaperone mutant *spt16–11* that depend on their histone methyltransferase activities and the Set2-Rpd3S pathway. Each AID mutant was transformed into YYW010 *(ΔSET2*), YYW163 (*spt16–11/ ΔSET2*) or YYW165 (*spt16–11 /ΔSET2/ΔRCO1*). Transformants were grown at 26°C until saturation. Five-fold serial dilutions of each transformant were then spotted on two plates and grown at 26 and 35°C. Photographs of the plates were taken after 2–3 days.

We next investigated the histone modification changes caused by AID mutations at a specific genomic locus. Consistent with the bulk level changes, ΔB led to slightly increased K36me3 while maintaining the same level of K36me2 at the *STE11* region (Figure 6A). Despite a lower expression level, ΔC caused elevated levels of both K36me2 and K36me3. Interestingly, ΔE gave rise to a higher level of K36me2 at *STE11* but lower K36me3 (Figure 6A). Therefore, it appears that AID mutations overall result in higher K36 methylation at coding regions. We noted that AID mutations caused increased K36me2 and K36me3 at promoter regions (A and B regions of *STE11*) as well, although the functional consequence of this abnormal pattern is unknown.

Since K36me regulates the histone acetylation level through the Set2-Rpd3S pathway, we also examined acetylation changes in these mutants. As reported previously, we found that deletion of *SET2* caused increase AcH4 levels at the *STE11* coding region (Figure 6A, vector). Modest AcH4 changes were detected in AID mutants as well (Figure 6A). Northern blotting analysis also revealed a mild cryptic transcription phenotype in these AID mutants (Supplementary Figure S6A). To confirm subtle differences detected in Northern analysis, we tested these AID mutants in an *STE11-HIS3* reporter strain in which the functional His3 protein can only be produced when the cryptic promoter of *STE11* is activated. Therefore, a defective Set2-Rpd3S pathway will allow cells to grow on histidine-depleted media (Supplementary Figure S6B). Indeed, we detected subtle cryptic transcription phenotypes in these mutants that were highly consistent with the Northern results (Supplementary Figure S6C). The severity of the phenotype among all mutants seemed to correlate with the levels of H3K36me3. Despite this cryptic transcription phenotype being very mild, this result is counter-intuitive considering that AID mutations cause hyperactive Set2. One possible cause of this surprising phenotype might be attributed to the instability of these AID mutant proteins. Previous studies suggested that Set2-mediated K36me not only helps Rpd3S bind to nucleosomal substrates but also involves activating Rpd3S to deacetylate neighboring nucleosomes (37–39). Despite these AID mutants being hyperactive, their retention time at coding regions, which is dictated by their association with elongating Pol II, might be shorter due to their fast turnover (Supplementary Figure S4). Therefore, it is possible that when Rpd3S travels through the regions and requires the K36me signal to function, mutant Set2 is prematurely degraded, thereby causing momentary defects.

We also took advantage of another genetic system to test the physiological roles of these AID mutants. Deletion of *SET2* or Rpd3S has been shown to rescue the growth of mutant *spt16–11*, a subunit of an essential histone chaperone known as FACT, at semi-permissive temperature (40,41). As expected, introducing these AID mutant plasmids to a Δ*SET2* strain did not result in any noticeable growth defect at 35°C (Figure 6B). However, when these AID mutants were expressed in *spt16–11/ΔSET2* yeast cells, we observed an interesting growth pattern at non-permissive temperature (35°C). AID mutations appeared to make cells grow even slower than when wild-type Set2 was introduced (Figure 6C). Given that it is the K36me that causes the lethality of *spt16–11* mutant, this result supported our conclusion above that AID mutations caused hyperactive Set2. We further demonstrated that this ‘sickness’ of AID/*spt16–11* mutants can be rescued by deletion of *RCO1*, the essential subunit of Rpd3S (Figure 6D). We concluded that AID mutants exerted this particular function through their catalytic activities as catalytically inactive ΔB and ΔD mutants (C201A) did not display such synthetic interactions (Figure 6E). Therefore, we confirmed that AID mutations led to hyperactive Set2 in another genetic system.

## DISCUSSION

Many chromatin modifying enzymes directly bind to phosphorylated CTD and presumably travel along with elongating Pol II, such as histone acetyltransferases NuA4 and SAGA, the histone deacetylase Rpd3S, histone methyltransferases Set2 and COMPASS, and the histone ubiquitylase Rad6 (37,42,43). The interactions between these factors and Pol II CTD have been well documented. However, little is known about how they coordinate to achieve precise spatial and temporal controls, particularly for those modification events that need to take place in specific orders. And it appears that the recruitment to Pol II is the only regulatory step within this process. Set2, as a classical example of these Pol II associated factors, contains clearly defined CTD-binding domain and catalytic domain. It has been shown that phosphorylated Pol II CTD is required for full activity of Set2 by increasing Set2 retention time at coding regions and maintaining Set2 stability (22). In this study, we found that beyond this initial recruitment step, p-CTD plays additional roles in regulating Set2 activity. Our biochemical analysis revealed that Set2 is wired with a sophisticated internal interacting network to ensure that the methylation events only happen at the right time and place.

Two key regulatory domains: SRI and the newly identified AID, work in concert to control the catalytic SET domain. We showed that besides binding to Pol II, SRI also recognizes the linker DNA of chromatin (Figure 3) and has the ability to prevent Set2 from methylating free histones (Figure 4D, SET-SRI), which are two important features that contribute to Set2 substrate specificity. Remarkably, a single SRI can not only stimulate Set2 activity on nucleosomes (Figure 4C, SET-SRI), which can be explained by its affinity to linker DNA, but can also inhibit Set2 activity on core histones (Figure 4D). Additionally, SRI can antagonize the repression activity that AID imposes on SET. As for the novel AID that we identified in this study, it can limit the SET domain activity. AID can also bind to unphosphorylated CTD (Figure 5F and Supplementary Figure S5C), which could potentially coordinate with the SRI-pCTD interaction and target Set2 to the specific region of CTD where phosphorylated and unphosphorylated CTD heptad repeats are adjacent to each other. Moreover, AID is also important for Set2 stability *in vivo* (Supplementary Figure S4). Since most of these AID mutants are stably folded and highly active when they are expressed in an insect cell system, we speculate that their instability may be not due to commonly interpreted mis-folding of mutant proteins. We showed that the CTD-Set2-FL fusion cannot function properly without CTD phosphorylation (Figure 1G and H). In this scenario, CTD contact is not essential for Set2 stability and p-CTD peptide does not stimulate Set2 HMT activity *in vitro* (20). Therefore, we suspect that p-CTD mediated Set2 targeting (Figure 1H) may be important for situating Set2 with unknown partners that regulate its activity and stability. AID mutations may disrupt such interactions, which could lead to the observed fast turn-over. Lastly, AID seems to fine-tune the interaction between SET and SRI. In the SET-SRI mutant, strong and mutual influences of these domains are obvious: SRI blocks SET free histone activity (Figure 4D) while SET inhibits the binding of SRI to p-CTD (Supplementary Figure S5C). However, in the presence of AID, these interplays are dampened. For instance, HMT activity of the ΔB mutant on core histones is higher than SET-SRI but lower than SET (Figure 5C). Without the B motif, AID cannot block SET histone activity. Thus, the repression activity stems from SRI within ΔB and it is weaker than when SRI can freely access SET.

Emerging lines of evidence suggest that auto-inhibition is a prevalent regulatory mechanism among chromatin regulators. This tight ‘on-site’ repression appear to be important to prevent uncontrolled activation and enable prompt responses to cellular stimuli (44). ISW1 chromatin remodeler contains two AIDs that suppress its adenosine triphosphate (ATP) hydrolysis and the coupling of ATP hydrolysis to DNA translocation without nucleosomal substrates. Nucleosomes provide two activating epitopes to antagonize AID, which then leads to productive chromatin remodeling (45). Histone methyltransferase Set1 is also tightly controlled by auto-regulation, in which balanced acts of an AID and an anti-inhibitory region dictate the enzyme activity (32,33). Interestingly, a recent study showed that Set1 protein level is also controlled by a feedback mechanism that senses productive H3K4 methylation and ongoing transcription (46). Likewise, Swi6, the heterochromatin protein 1 (HP1) in fission yeast, was shown to transit from an auto-inhibited state to a spreading-competent state upon contact with an H3K9 methyl mark and nucleosomal DNA (47). In our study, we found that the AID of Set2 is not only important for establishing Set2 substrate specificity but also for influencing its stability and physiological functions. Set2 is a highly conserved histone methyltransferase family. Setd2, the human homolog of yeast Set2, also contains the middle region that shares a similar predicted secondary structure to AID as we reported here. Importantly, Setd2 is one of the most frequently mutated genes among chromatin regulators in various tumor types (48). Many disease-linked mutations are missense mutations that span the entire protein, including the potential auto-inhibitory region. Therefore, our mechanistic study here might provide important insights into how those mutations disrupt Setd2 tumor suppressor functions.

## SUPPLEMENTARY DATA

Supplementary Data are available at NAR Online.

SUPPLEMENTARY DATA
