# Supplementary material for: Balancing acts of SRI and an auto-inhibitory domain specify Set2 function at transcribed chromatin
Source: Nucleic Acids Res. 2015 Apr 29;43(10):4881–92. doi: 10.1093/nar/gkv393 (PMC4446442; doi:10.1093/nar/gkv393)
Supplement: SUPPLEMENTARY DATA [file supp_43_10_4881__index.html]

Balancing acts of SRI and an auto-inhibitory domain specify Set2 function at transcribed chromatin — Balancing acts of SRI and an auto-inhibitory domain specify Set2 function at transcribed chromatin — SUPPLEMENTARY DATA 

# Balancing acts of SRI and an auto-inhibitory domain specify Set2 function at transcribed chromatin

## SUPPLEMENTARY DATA

**Files in this Data Supplement:**

- SUPPLEMENTARY DATA
