# Supplementary material for: Balancing acts of SRI and an auto-inhibitory domain specify Set2 function at transcribed chromatin
Source: Nucleic Acids Res. 2015 Apr 29;43(10):4881–92. doi: 10.1093/nar/gkv393 (PMC4446442; doi:10.1093/nar/gkv393)
Supplement: SUPPLEMENTARY DATA [file supp_gkv393_nar-03476-x-2014-File008.pdf]

Balancing acts of SRI and an auto-inhibitory domain specify Set2 function at transcribed chromatin

Yi Wang and Bing Li*

Biological Chemistry Graduate Program, UT Southwestern Medical Center, Dallas, TX, USA
Department of Molecular Biology, UT Southwestern Medical Center, 5323 Harry Hines Blvd., Dallas, TX 75390

*Correspondence to: Bing4.Li@utsouthwestern.edu

Supplementary Data

Materials and Methods

Construction of Plasmids and Yeast Strains:

Plasmids and yeast strains were constructed through standard procedures and are listed in Tables S1 and S2, respectively.

To engineer a TEV cleavage site into Set2, two fragments of DNA were prepared separately as illustrated in Figure 4B. One fragment contained the SET domain of Set2 flanked by the 5' XhoI site and the 3'end BamHI site plus the TEV-recognition site and a flexible linker. The other fragment included the rest of the C-terminal portion of Set2 (or different truncations) with a 5'BamH I site and a 3' NotI site. Those two fragments were ligated through the BamHI site and subcloned into XhoI/Not I sites of pBL532. To generate deletion mutants at the middle of Set2 (Figure 6A), similar approaches were taken. In this case, the 5' fragment contained the sequence corresponding to the N-term pieces before the break point with a 3' BamHI site, while the 3' fragment covered the C-terminal part of Set2 with a 5' BamHI site. To construct pWY084, a vector for expressing *SET2* under the control of the *ADH1* promoter, an adaptor containing a FLAG tag sequence and XhoI/NotI restriction sites was inserted into pWY080 (a gift from Dr. Brian Strahl). All Set2 mutant fragments from the baculovirus system were directly subcloned into the pWY084 through the same XhoI/Not I sites.

To construct Rpb1-Set2 fusion plasmids, an intermediate vector, pBL875, was first constructed. The KpnI/SnaBI fragment at the *RPB1* region was subcloned into a modified pBluescript vector, pBL766. A linker containing two restriction sites (SmaI and XhoI) and the sequence for an HA tag was inserted between the last codon of the heptad repeat sequence and the 3' UTR of RPB1. The coding sequences of *SET2* and its derivatives were then subcloned into the XhoI site of pBL875 to form pWY041 (full length Set2; FL), pWY042 (coding for Set2 residues 1-618) and pBL876 (SET domain). The KpnI/SnaBI fragments from these intermediate vectors were subsequently transferred into pBL855 (pY1A) to generate pWY043 (CTD-Set2 FL), pWY044 (CTD-Set2 1-618), pWY045 (CTD-Set2 SET) and pWY046 (CTD-HA, from the empty vector pBL875). The resulting constructs were shuffled into YYW120 (Z26) and YYW121 (Z26, Δ*SET2*) using the standard 5'-FOA method so that Pol II-Set2 fusions are the only source of RNA polymerase II in the final strains.

To make the YYW031(*HIS3::*p*GAL1*-3HA-Set2 */ΔCDC73::KAN*) and YYW032 (*HIS3::*p*GAL1*-3HA-Set2 */ΔPAF1::KAN*) strains, YYW026-A(*HIS3::*pGAL1-3HA-Set2, MATa) was crossed with YBL703 (*ΔCDC73::KAN* MAT alpha) and YBL701 (*ΔPAF1::KAN* MAT alpha), respectively. Selected diploid cells were seeded in sporulation media containing 1% potassium acetate for about 1 week due to inefficient sporulation of these mutants. Spores were dissected and the presence of *HIS* and *KANMX* cassettes was screened by PCR. Lastly, western blotting with anti-HA antibody was performed to confirm proper expression of HA-tagged Set2 in dextrose- and galactose-containing media.

Preparation of whole-cell extracts for immunoblotting:

Cells were grown in 3 ml YPD or synthetic drop-out media supplemented with 2% dextrose or 2% galactose at 30°C overnight until the OD_{600} reached 1.0-1.2. Cell pellets were re-suspended in 45 μl of STE buffer (500 mM NaCl, 10 mM Tris HCl pH8.0 and 1 mM EDTA) and 40 μl of 3xSDS buffer. 100 μl of 0.5 mm glass beads were then added and boiled at 95°C for 5 min. Samples were then vigorously vortexed to break the cells, and cell suspensions were clarified by spinning at 14,000 rpm for 5 min. Anti-H3K36me3 (Abcam, 9050), anti-H3K36me2 (Abcam, 9049), anti-H4 (Abcam, 10158) and anti-FLAG HRP (Sigma, A8592) antibodies were used according to the manufacturers' instructions. For anti-H3K36me2 antibody, 5% BSA was used for blocking and 1% BSA was present in all antibody incubations.

Protein Purification:

Insect cell system: 100 ml Sf21 cells ($1X10^6$ cells/ml) supplemented with 10% FBS (Sigma), 1% Penicillin Streptomycin (Life Technologies) were infected with approximately 10 ml of each P2 virus and incubated at 27°C for 48 hours (1). Cells were collected and washed with 10 ml 1X PBS buffer. Cell pellets were then lysed in 10 ml BV-lysis buffer (50 mM HEPES PH7.9, 500 mM NaCl, 10% glycerol,0.5 mM EDTA, 2 mM $MgCl_2$ and 0.2% Triton X-100) on ice for 30 min. The lysates were clarified by ultracentrifugation (Beckman 50.2 Ti 40,000 rpm) for 30 min. 200 µl of Anti-Flag M2 affinity beads (Sigma) was added to the supernatants and incubated at 4°C for at least 2 hours. Beads were then washed with 10 ml BV-lysis buffer 3 times and eluted with 600 µl of 500 µg/ml of 3xFLAG peptides (Sigma F4799) in BV elution buffer (50 mM HEPES pH7.9; 100 mM NaCl; 2 mM $MgCl_2$; 0.02% NP40 and 10% glycerol). The eluents were concentrated to about 150 µl using 10 kd cut-off concentrators (Amicon).

TAP purification from yeast: TAP purification was performed essentially as described in our previous publication (2). Briefly, 6 liters of TAP-tagged Set2 derivative strains were grown in YPAD media supplemented with 2% dextrose at 30°C OD_{600}=2-3. The cell pellets were re-suspended in the Extraction buffer (E buffer) (40 mM HEPES pH7.5, 350 mM NaCl, 10% glycerol, 0.1% Tween 20 and protease inhibitor cocktail (Sigma P8215) and disrupted by bead beating (Biospec). Crude whole cell extracts were clarified by ultracentrifugation (Beckman 50.2 Ti at 45,000 rpm) and then incubated with 600 µl IgG-Sepharose (GE) at 4°C overnight. Bound proteins were cleaved from the beads using 100 units of TEV protease (Invitrogen) at 18°C for 3 hours. The eluents were mixed with 3 ml of Calmodulin Binding Buffer (10 mM Tris.HCl pH8.0, 150 mM NaCl, 1 mM magnesium acetate, 1 mM imidazole, 2 mM $CaCl_2$, 10 mM β-ME, 0.1% NP40 and 10% glycerol) and 3 µl of 1 M $CaCl_2$. They were then incubated with 600 µl Calmodulin-Sepharose (GE) at 4°C for 1 hour. The bead-bound proteins were finally eluted by Calmodulin Elution Buffer (10 mM Tris.HCl pH8.0, 150 mM NaCl, 1 mM magnesium acetate, 1 mM imidazole, 2 mM EGTA pH8.0, 10 mM β-ME, 0.1% NP40 and 10% glycerol) and concentrated using Amicon concentrators.

Recombinant Histone Purification and Nucleosome Reconstitution

Xenopus recombinant histones (H3, H4, H2A and H2B) were individually expressed in

BL21CodonPlus-RIL (Stratagene) cells and purified as described (3). Histone octamers were assembled and fractionated through gel-filtration column Superdex 200. To generate mono-nucleosomes with different linker DNA lengths, plasmids that carry 16 copies of 216 bp (pBL645), 153 bp (pBL647) or 147 bp (pBL648) DNA fragments were digested by EcoRV, and the template DNA were purified using the 491 Prep-cell (Bio-Rad) (4). These DNA fragments were then mixed with histone octamers at a pre-determined ratio in high salt buffer and dialyzed against a buffer with serial dilutions of salt concentrations (4). The reconstituted nucleosomes were finally purified through a 4% native gel using 491 Prep-cell (Bio-Rad) and concentrated to about 1 μg/μl (4).

Yeast native nucleosome purification

Yeast nuclei were prepared as previously described (5) with minor modifications. Briefly, 3 liters of YYW010 *(ΔSET2)* cells were cultured to OD_{600}=1.0. Cell pellets were re-suspended in cold SB buffer (40 mM HEPES.NaOH pH7.5, 0.5 mM $MgCl_2$, 1.4 M sorbitol, 10 mM βME and 1 mM PMSF) and digested with Zymolyase (Seikagaku 100T) for up to 30 min at 30°C. The spheroplasts were washed with SB buffer twice and resuspended in cold FB buffer (20 mM PIPES.NaOH pH6.5, 0.5 mM $MgCl_2$ and 18% Ficoll 400) and homogenized using a Thomas Pestle Tissue Grinder (Size C). The resulting suspension was laid over 10 ml GB buffer (20 mM PIPES.NaOH pH6.5, 0.5 mM $MgCl_2$, 7% Ficoll 400 and 20% glycerol) and centrifuged at 11,500 rpm using a HB6 swinging-bucket rotor. The pellets were resuspended in 20 ml of FB buffer, and spun at 4,500 rpm to remove cell debris. The nuclei were finally collected by centrifugation at 11,500 rpm for 30 min, and extracted three times with 120 ml of EBX buffer (50 mM HEPES.NaOH pH7.5, 2.5 mM $MgCl_2$, 400 mM KCl and 0.25% Triton X-100). The remaining chromatin pellets were resuspended in 10 ml MNase digestion buffer (10 mM HEPES.NaOH pH7.5, 0.5 mM $MgCl_2$ and 0.05 $CaCl_2$) and sonicated briefly to break the clumps. After a small-scale MNase titration test, the optimal concentration of Micrococcal nuclease (MNase) was applied to the rest of the chromatin suspension at 30°C for 15 min. MNase digestion was stopped by addition of EDTA. The digested oligonucleosomes were then fractionated on a Sepharose CL-6B column using 0.1 M HB buffer (20 mM HEPES.NaOH pH7.5, 1 mM EDTA, 100 mM NaCl, 10% glycerol and 1 mM βME). Nucleosomes were pooled based on the size of DNA and concentrated using 30 kd cutoff concentrators (Amicon).

CTD peptide pull-down Assay:

Biotinylated peptides corresponding 4 heptad repeats of unphosphorylated (CTD), or Serine2 phosphorylated (Ser2P) or Serine 5 phosphorylated (Ser5P) Pol II CTD were immobilized on Dynabeads® M-280 Streptavidin (Invitrogen) at 4°C for 2 hours. The beads carrying 8 µg of each peptide were used in each pull-down reaction and resuspended in 50 µl of Peptide Binding Buffer (25 mM Tris.HCl pH 8.0, 50 mM NaCl, 5% glycerol, 0.03% NP40 and 1 mM DTT). 1 µg full-length Set2 or an equal molar amount of mutant proteins was used for each reaction. The bound proteins were detected by western blots using anti-Flag HRP (Sigma) or anti-GST (Santa Cruz) antibodies.

Northern Blotting Assay:

Total RNA was extracted by vortexing cells in 200 µl of 0.5 mm glass beads (Biospec), 320 µl of RNA Prep Buffer (0.5 M NaCl, 0.1 M Tris-HCl pH 7.5, 10 mM EDTA and 1% SDS) and 250 µl of Phenol-Chloroform Isoamyl-alcohol (Fisher). RNAs were then ethanol precipitated, resolved on 1.4% agarose-formaldehyde gels and transferred onto a Zeta-probe membrane (Bio-Rad). Northern blotting was carried out in Denhardt's based buffer (6X SSC, 5X Denhardt's solution, 0.5% SDS and 0.1 mg/ml of sonicated salmon sperm DNA). 3' *STE11* probe was prepared through an in vitro transcription reaction using T7 RNA polymerase (MEGAscript® Kit, Life Technologies Corporation) and 5 µl of α-^{32}P labeled CTP (Perkin Elmer).

Supplementary Figures

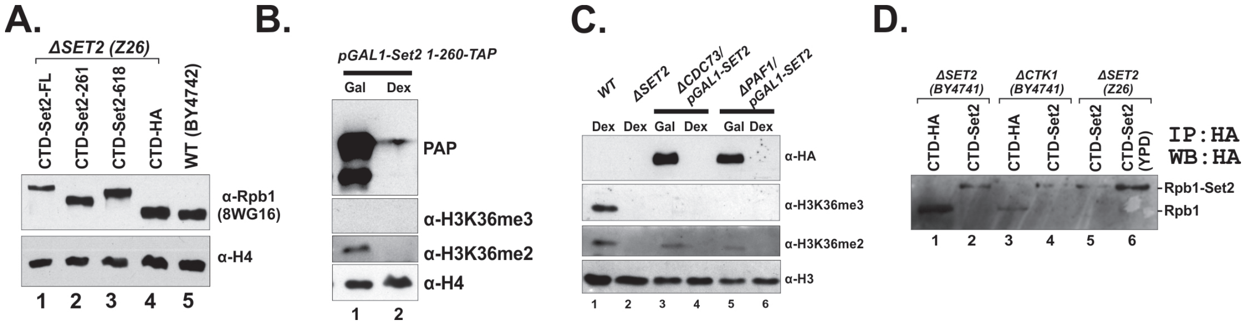

Figure S1. Binding to phosphorylated Pol II CTD is essential for Set2-mediated H3K36me3.

(A) Stable expression of the Set2 derivatives fused to the C-terminus of Rpb1. Western blot of Rpb1-Set2 fusion proteins as described in Figure 1B. Slightly reduced level of CTD-Set2-FL might be due to the inefficient transfer of the high molecular weight protein. (B) Overexpression of the Set2 SET domain alone does not result in K36me3. Western blots analysis of whole cell extracts from YYW042 (*HIS3:: pGAL1-3HA-Set2 1-260-TAP::URA3*) grown in YP medium containing 2% galactose (Gal) or dextrose (Dex). (C) The PAF.com is required for K36me3 even when Set2 is overexpressed. Western blots of cell extracts from YYW031 (*HIS3::* pGAL1-*3HA-Set2 /ΔCDC73::KAN*) and YYW032 (*HIS3::pGAL1-3HA-Set2 /ΔPAF1::KAN)* grown in YP medium containing galactose or dextrose. (D) Fusing to Rpb1 stabilizes Set2 in Δ*CTK1*. Yeast whole cell extracts were prepared from yeast strains grown in either SD-Leu (Lane 1-5) or YPD (Lane 6), the anti-HA resins were then added to immunoprecipitate HA-tagged Rpb1-fusion proteins. All bound proteins were eluted in SDS-PAGE loading buffer and detected by western blotting.

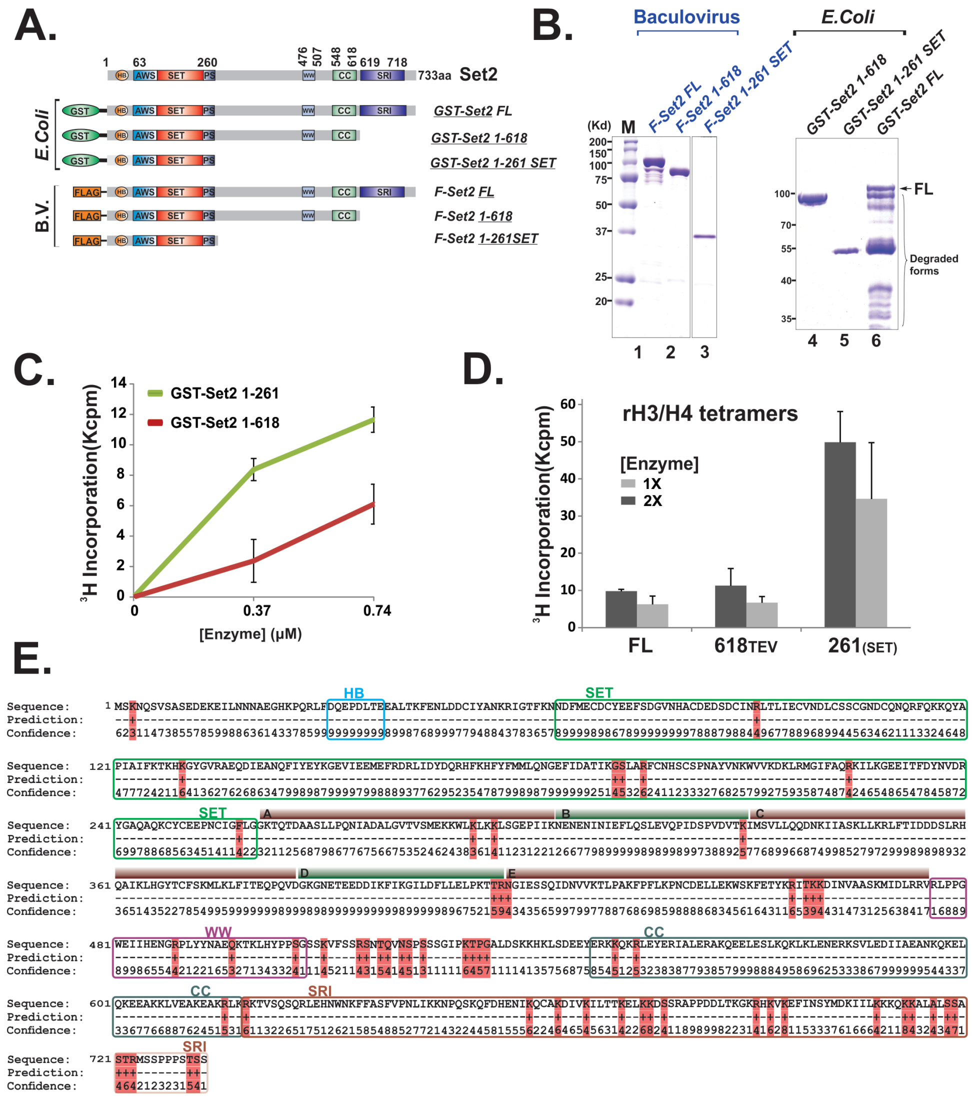

Figure S2. Full-length Set2 and SET domain have distinct substrate preferences.
(A) An illustration of recombinant Set2 and truncated derivatives. The SET domain, histone binding motif (HB), AWS domain, post SET domain (PS), WW domain, coiled-coil motif (CC) and Set2-Rpb1 interaction domain (SRI) are shown along with their positions within full-length Set2. (B) Coomassie staining of Set2 and mutants purified from a baculovirus expression system and E. coli. (C) Standard HMT assay using HeLa oligonucleosome substrates showing the GST-Set2-618 is in a repressed state. (D) HMT assay using *Xenopus* H3/H4 tetramers as substrates. (E) The residues of Set2 bind to DNA as predicted by the BindN+ (http://bioinfo.ggc.org/bindn+/) webserver. Predicted DNA binding residues are labeled with '+' and labeled in red. Non-binding residues are marked with '-'. Confidence factors are listed underneath. Key domains of Set2 are highlighted in different colors.

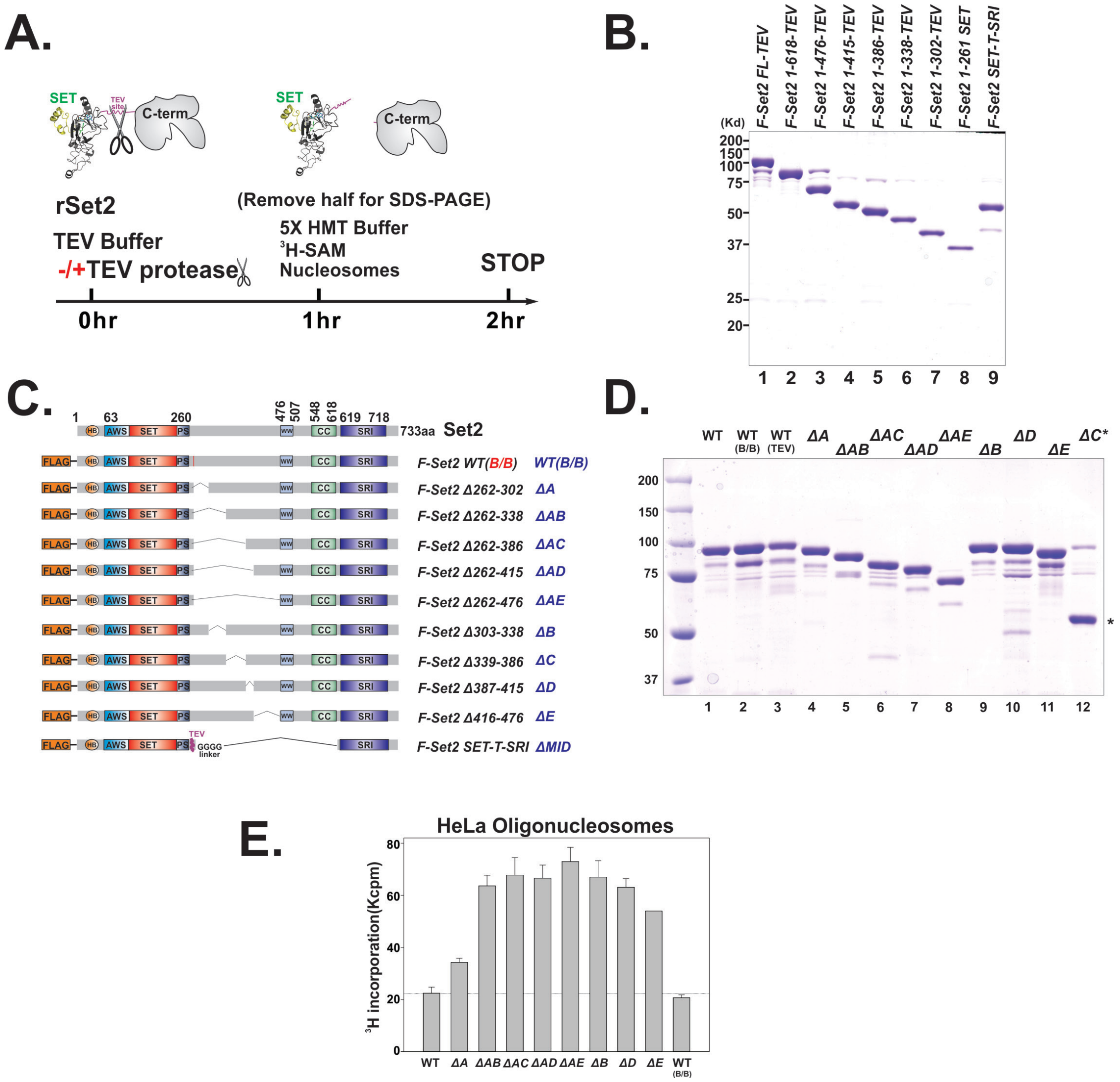

Figure S3. Full-length Set2 and SET domain have distinct substrate preferences.
(A) An experimental strategy to test if SET activity is intrinsically inhibited. (B) Coomassie staining of Set2 TEV proteins that were purified from insect cells as indicated in Figure 4A. (C) Set2 constructs for mapping minimal deletion that causes de-repression of SET activity. (D) Coomassie staining of purified Set2 proteins. The asterisk indicates the degraded protein. Note that Set2 ΔC mutant purified from insect cell expression system consistently displayed severe degradation. (E) Integrity of the AID domain is essential for regulating Set2 activity in vitro as measured by HMT assays using HeLa oligonucleosome substrates as described in Figure 5B.

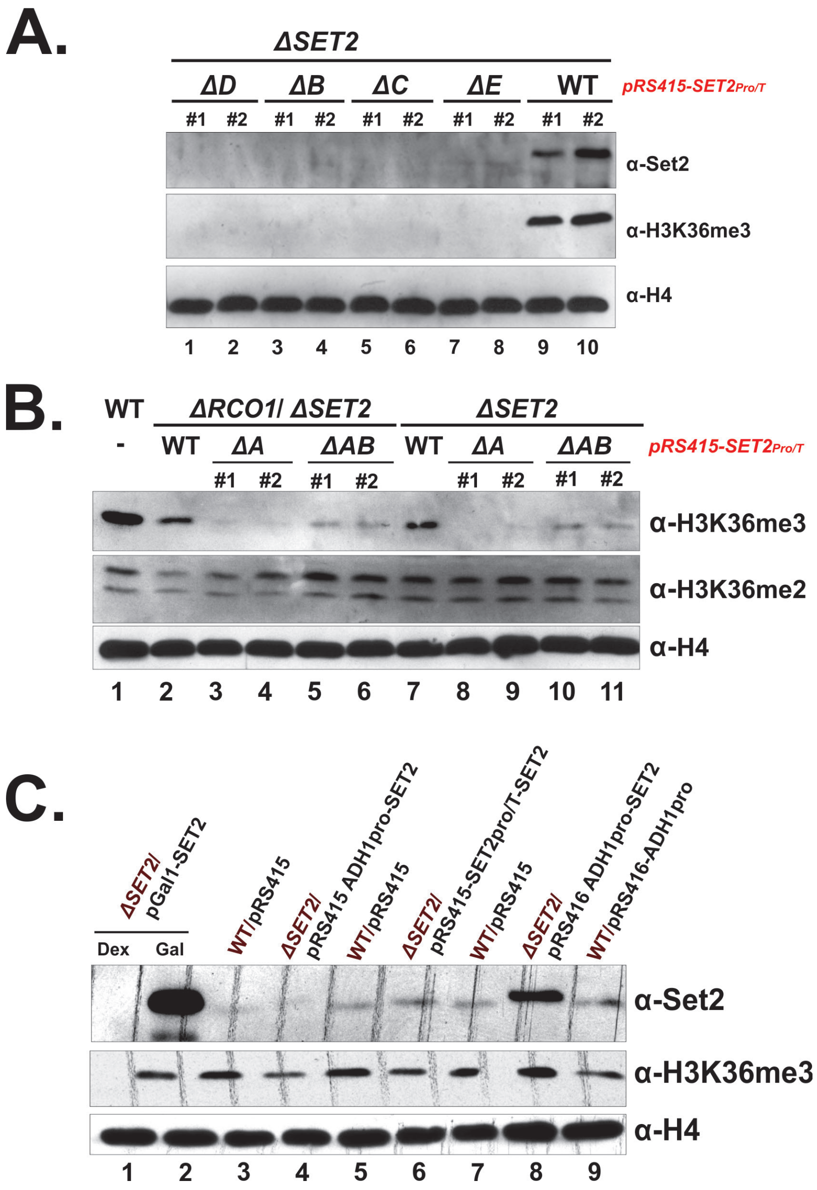

Figure S4. AID mutations render Set2 more sensitive to degradation

Western blots of whole cell extracts from the indicated yeast strains. #1 and #2 represent two independent clones of the same strain that were tested. (A) Plasmids carrying WT or AID-mutated Set2 under the control of native *SET2* promoters were transformed into YYW010 (Δ*SET2*). (B) Deletion of *RCO1* does not alleviate H3K36 status changes caused by AID mutations. pWY126 and pWY127 were transformed into YYW132(Δ*SET2*/Δ*RCO1*). Western blotting was performed with the indicated antibodies. (C). Protein levels of Set2 and H3K36 methylation status in strains in which *SET2* is under the control of different promoters: pWY069 (*GAL1*) and pWY131 (*SET2* native promoter). Two plasmids containing *ADH1* promoter, pWY088 (Ura3) and pWY091 (Leu2), were transformed into Δ*SET2*. Their empty vector counterparts were transformed into WT strain. Western blotting was performed with anti-Set2 antibody (a generous gift from Dr. Strahl) to detect the expression levels of plasmid-borne Set2 compared to the endogenous source.

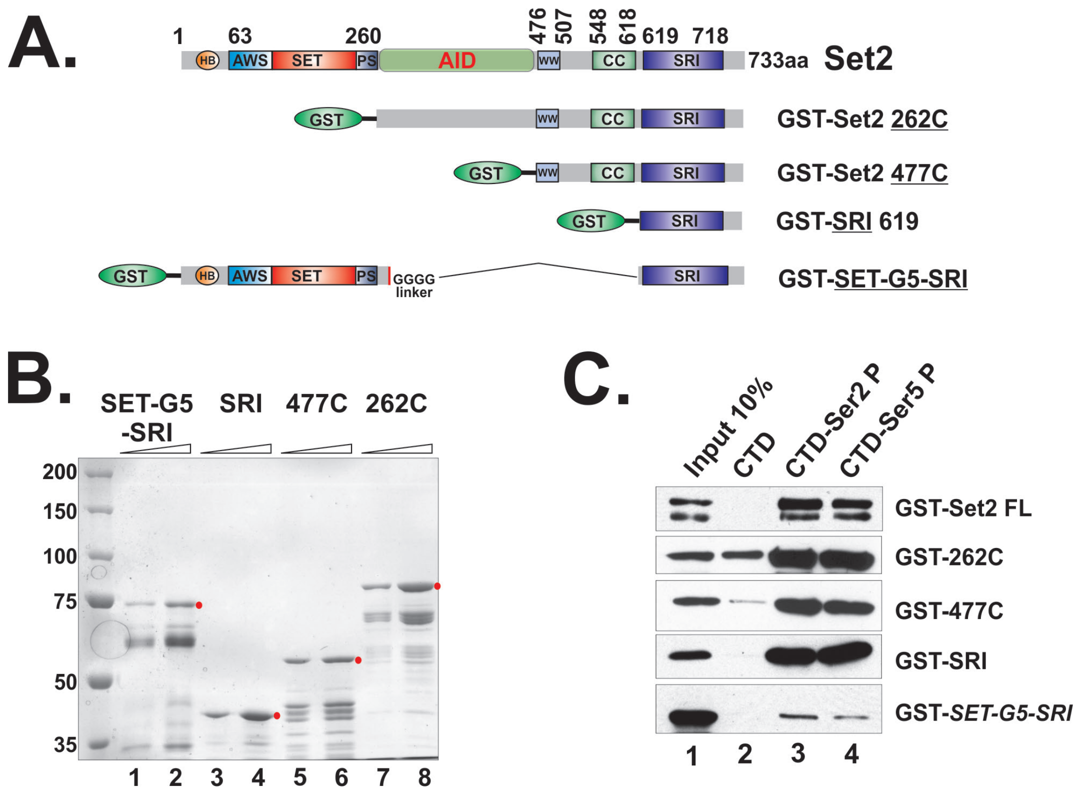

Figure S5. AID regulates the binding of Set2 to Pol II CTD
(A) A schematic illustration of constructs for GST versions of FL Set2 and its N-terminal truncations. The SET-G5-SRI was constructed by fusion of the SET and SRI domains with a five poly-glycine linker. (B) The Coomassie staining of purified GST-Set2 variants. (C) CTD pull-down assay using recombinant GST Set2 variants.

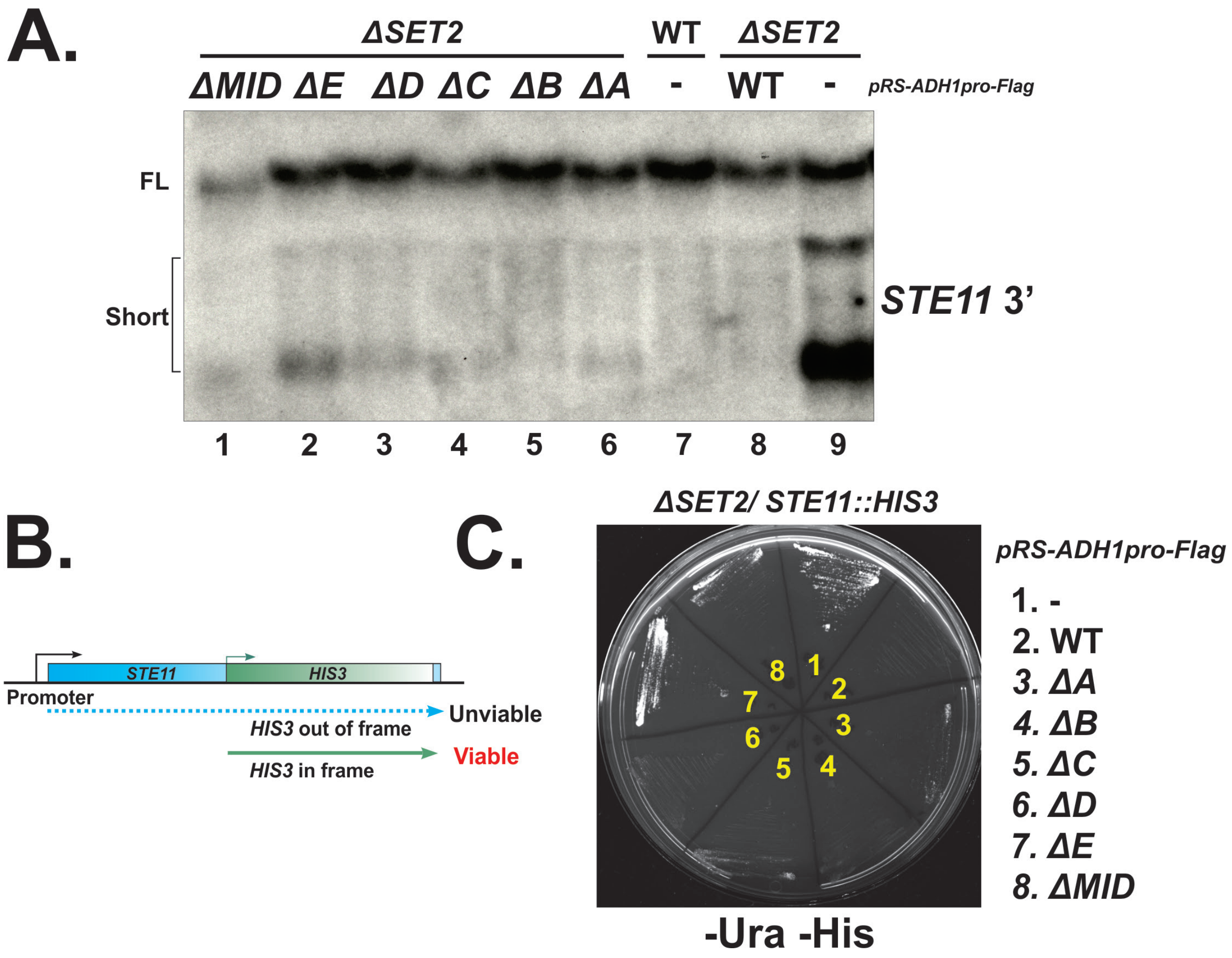

Figure S6. AID mutations lead to cryptic transcription phenotype
(A) Total RNA from the indicated yeast strains were subjected to Northern blots using *STE11*-3'RNA probe that was labeled through in vitro transcription. Full length and two short transcripts of *STE11* are indicated. (B) A diagram of cryptic transcription reporter strains in which HIS3 genes were integrated downstream of the *STE11* cryptic promoter (*STE11-HIS3*). The integration sites were selected such that the *HIS3* gene is out of frame with relation to the *STE11* coding region, and the functional His3 can only be produced when the *HIS3* transcript initiates at the cryptic promoter of *STE11*. (C) Indicated plasmids were transformed into YCR377 (*STE11-HIS3*/Δ*SET2*). The resulting strains were grown on SD-URA/-HIS plates at 30°C for 5 days.

Supplementary Tables

Table S1. List of plasmids.

Plasmid	Backbone	Description	Source
**pBL196**		*pRET-N-GST*	Li Lab
**pBL532**	pBP-HFT	*pBacPAK-8-N-HisFlag-TEVsite*	Li Lab
**pBL645**	**pBS-105**	***pBS-216L-16X***	Li Lab
**pBL647**	**pBS-305**	***pBS-153C-16X***	Li Lab
**pBL648**	**pBS-405**	***pBS-147-16X***	Li Lab
**pBL766**	pBL765	*pBSKO-NgoM4-Bts1-2-3*	Li Lab
**pBL773**	pBL766	*pBSaK-Rpb1-KSn-Linker*	Li Lab
**pBL855**		*pYIA*	John Stiller
**pBL869**	pBL196	*pRET-N-GST-XhoI-NotI*	Li Lab
**pBL870**	pCR-Blunt	*pCR-B-CTD-fusion-Up*	Li Lab
**pBL871**	pCR-Blunt	*pCR-B-CTD-fusion-Down*	Li Lab
**pBL872**	pCR-Blunt	*pCR-B-yeast Set2-SET (Xho1) for CTD fusion*	this study
**pBL875**	pBL733	*pBSaK-KSn-CTD fusion (Xho1) HA*	this study
**pBL876**	pBL875	*pBSaK-KSn-CTD fusion Set2-SET(Xho1)-HA*	this study
**pWY001**	pBL196	*pRET-GST-Set2 1-261*	this study
**pWY001/A**	pBL196	*pRET-GST-Set2*	this study
**pWY005**	pBL532	*pBP-HFT-Set2*	this study
**pWY037**	pBL532	*pBP-HFT-Set2 1-261*	this study
**pWY038**	pBL532	*pBP-HFT-Set2 1-618*	this study
**pWY039**	pCR-Blunt	*pCR-B-Set2 FL (Xho1) for CTD fusion*	this study
**pWY040**	pCR-Blunt	*pCR-B-Set2 1-618 (Xho1) for CTD fushion*	this study
**pWY041**	pBL875	*pBSaK-KSn-CTD-fusion-Set2 FL(Xho1)-HA*	this study
**pWY042**	pBL875	*pBSaK-KSn-CTD-fusion-Set2 1-618(Xho1)-HA*	this study
**pWY043**	pBL855	*pYIA CTD-fusion-Set2 (KpnI/SnaBI)*	this study
**pWY044**	pBL855	*pYIA CTD-fusion-Set2 1-618 (KpnI/SnaBI)*	this study
**pWY045**	pBL855	*pYIA CTD-fusion-Set2 1-261 (KpnI/SnaBI)*	this study
**pWY046**	pBL855	*pYIA CTD-fusion(KpnI/SnaBI)*	this study
**pWY050**	pBL532	*pBP-HFT-Set2 1-618 TEV*	this study
**pWY051**	pBL532	*pBP-HFT-Set2 1-476 TEV*	this study
**pWY061**	pBL532	*pBP-HFT-Set2 SET-G5-SRI TEV (Δ262-618)*	this study
**pWY062**	pBL532	*pBP-HFT-Set2 1-302 TEV*	this study
**pWY063**	pBL532	*pBP-HFT-Set2 1-338 TEV*	this study
**pWY064**	pBL532	*pBP-HFT-Set2 1-386 TEV*	this study
**pWY065**	pBL532	*pBP-HFT-Set2 1-415TEV*	this study
**pWY066**	pBL532	*pBP-HFT-Set2 1-733 TEV (FL)*	this study
**pWY069**	pBL354	*pRS415-pGAL1-HA-Set2*	this study
**pWY080**	pRS416	*pRS416-ADH1pro-Set2-CYC1ter*	Brian Strahl

**pWY084**	pWY080	*pRS416-ADH1pro-ADPT2-FLAG-CYC1ter*	this study
**pWY087**	pWY084	*pRS416-ADH1pro-FLAG-Set2 SET-G5-SRI-TEV(Δ262-618)*	this study
**pWY088**	pWY084	*pRS416-ADH1pro-FLAG-Set2 FL*	this study
**pWY091**	pRS415	*pRS415-ADH1pro-ADPT2-FLAG-Set2FL-CYC1ter*	this study
**pWY101**	pBL532	*pBP-HFT-Set2 FL(BglII/BamHI) (B/B)*	this study
**pWY102**	pBL532	*pBP-HFT-Set2 ΔAB (Δ262-338)*	this study
**pWY103**	pBL532	*pBP-HFT-Set2 ΔA-C (Δ262-386)*	this study
**pWY104**	pBL532	*pBP-HFT-Set2 ΔA-E (Δ262-476)*	this study
**pWY105**	pBL532	*pBP-HFT-Set2 ΔB (Δ303-338)*	this study
**pWY106**	pBL532	*PBP-HFT-Set2 ΔC (Δ339-386)*	this study
**pWY107**	pBL532	*pBP-HFT-Set2 ΔE (Δ416-476)*	this study
**pWY120**	pBL532	*pBP-HFT-Set2 ΔA (Δ262-302)*	this study
**pWY121**	pBL532	*pBP-HFT-Set2 ΔA-D (Δ262-415)*	this study
**pWY122**	pBL532	*pBP-HFT-Set2 ΔD (Δ387-415)*	this study
**pWY123**	pBL196	*pRET-GST-Set2-SET-G5-SRI*	this study
**pWY126**	pWY074	*pRS415-Set2 Pro/Ter-HA-Set2 ΔA (Δ206-303)*	this study
**pWY127**	pWY074	*pRS415-Set2 Pro/Ter-HA-Set2 ΔAB (Δ206-338)*	this study
**pWY128**	pBL196	*pRET-GST-Set2-SRI*	this study
**pWY129**	pWY084	*pRS416-ADH1pro-FLAG-Set2 ΔA (Δ262-302)*	this study
**pWY130**	pWY084	*pRS416-ADH1pro-FLAG-Set2 ΔAB (Δ262-338)*	this study
**pWY131**	pBL944	*pRS415-Set2 Pro/Ter-HA-Set2*	this study
**pWY136**	pWY084	*pRS416-ADH1pro-FLAG-Set2 ΔC (Δ339-386)*	this study
**pWY137**	pWY084	*pRS416-ADH1pro-FLAG-Set2 ΔB (Δ303-338)*	this study
**pWY138**	pWY084	*pRS416-ADH1pro-FLAG-Set2 ΔD (Δ387-415)*	this study
**pWY139**	pBL196	*pRET-GST-Set2-477C*	this study
**pWY141**	pBL196	*pRET-GST-Set2-262C*	this study
**pWY151**	pWY084	*pRS416-ADH1pro-FLAG-Set2 ΔE (Δ416-476)*	this study
**pWY152**	pWY084	*pRS416-ADH1pro-FLAG-Set2 ΔA-E (Δ216-476)*	this study
**pWY182**	pWY138	*pRS416-ADH1pro-FLAG-Set2 ΔD-C201A*	this study
**pWY184**	pWY137	*pRS416-ADH1pro-FLAG-Set2 ΔB-C201A*	this study
**pWY215**	pWY074	*pRS415-Set2 Pro/Ter-HA-Set2 ΔD (Δ387-415)*	this study
**pWY216**	pWY074	*pRS415-Set2 Pro/Ter-HA-Set2 ΔB (Δ303-338)*	this study
**pWY217**	pWY074	*pRS415-Set2 Pro/Ter-HA-Set2 ΔC (Δ339-386)*	this study
**pWY218**	pWY074	*pRS415-Set2 Pro/Ter-HA-Set2 ΔE (Δ416-476)*	this study
**pWY239**	pBL869	*pRET-GST-Set2 1-618*	this study

Table S2. List of yeast strains.

Name	Parental strain	Genotype	Source
YBL272	BY4741	*MATa his3Δ1 leu2Δ0 met15Δ0 ura3Δ0* ***ΔCTK1::KAN***	Li Lab
YBL360	BY4741	*MATa his3Δ1 leu2Δ0 met15Δ0 ura3Δ0* ***Rpb9-TAP****::HIS3*	Li Lab
YCR377	YCR376	*MATa his3Δ1 leu2Δ0 lys2Δ 0 ura3Δ0* ***STE11 1840-HIS3 ΔSET2::HPH***	Li Lab
YYW010	BY4742	*MATalpha his3Δ1 leu2Δ0 lys2Δ 0 ura3Δ0* ***ΔSET2::KAN***	Li Lab
YYW026/A	BY4741	*MATa his3Δ1 leu2Δ0 lys2Δ 0 ura3Δ0* ***HIS3::pGAL1-3HA-Set2***	this study
YYW031	YBL703x YYW026/A	*his3Δ1 leu2Δ0 lys2Δ 0 ura3Δ0* ***ΔCDC73::KAN HIS3::pGAL1-3HA-Set2***	this study
YYW032	YBL701x YYW026/A	*his3Δ1 leu2Δ0 lys2Δ 0 ura3Δ0* ***ΔPAF1::KAN HIS3::pGAL1-3HA-Set2***	this study
YYW037	PH499	*MATa ura3-52 lys2-801 ade2-101 his3-Δ200 leu2-Δ1****Set2-C-TAP::TRP1***	Li Lab
YYW040	BY4742	*MATalpha his3Δ1 leu2Δ0 lys2Δ 0 ura3Δ0* ***Set2 1-618-C-TAP::URA3***	this study
YYW042	BY4742	*MATalpha his3Δ1 leu2Δ0 lys2Δ 0 ura3Δ0* ***HIS3::pGAL1-3HA-Set2 1-260-C-TAP::URA3***	this study
YYW044	BY4742	*MATalpha his3Δ1 leu2Δ0 lys2Δ 0 ura3Δ0* ***Set2 1-260-C-TAP ::URA3***	this study
YYW045	YBL699	*MATa his3Δ1 leu2Δ0 met15Δ0 ura3Δ0* ***ΔRPH1::KAN ΔJHD1::Cre-LEU2 Set2 1-260-C-TAP::URA3***	this study
YYW087	BY4742	*MATalpha his3Δ1 leu2Δ0 lys2Δ 0 ura3Δ0* ***Set2-C-TAP::URA3***	this study
YYW114	YYW120	*MATalpha hisΔ200 ura3-52 leu2-3,112* ***rpb1Δ187:: HIS3 +pWY046(pYIA CTD-fusionKpnI/SnaBI Leu2)***	this study
YYW115	YYW120	*MATalpha hisΔ200 ura3-52 leu2-3,112* ***rpb1Δ187:: HIS3 +pWY045(pYIA CTD-fusion-SetT2 1-261KpnI/SnaBI Leu2)***	this study
YYW117	YYW121	*MATalpha hisΔ200 ura3-52 leu2-3,112* ***rpb1Δ187:: HIS3 ΔSET2::HPH+pWY046(pYIA CTD-fusion KpnI/SnaBI Leu2)***	this study
YYW118	YYW121	*MATalpha hisΔ200 ura3-52 leu2-3,112* ***rpb1Δ187:: HIS3 ΔSET2::HPH+pWY045(pYIA CTD-fusion-Set2 1-261KpnI/SnaBI Leu2)***	this study
YYW120	Z26	*MATalpha hisΔ200 ura3-52 leu2-3,112* ***rpb1Δ187:: HIS3 +pRP112 (RPB1 CEN URA3)***	John Stiller
YYW121	YYW120	*MATalpha hisΔ200 ura3-52 leu2-3,112* ***rpb1Δ187:: HIS3ΔSET2::HPH*** *+* ***pRP112 (RPB1 CEN URA3)***	this study
YYW122	CKY283	*MATa ura3-52 his3Δ200 leu2Δ1 or Δ0 trp1Δ63 met15Δ0 lys2-128 ∂ gal10Δ56 rpb1Δ::CLONATMX* ***Rpb3-TAP::KlacTRP1+pRP112 (RPB1 CEN URA3)***	C. Kaplan
YYW123	YYW120	*MATalpha hisΔ200 ura3-52 leu2-3,112* ***rpb1Δ187:: HIS3 +pWY044(pYIA CTD-fusion-Set2 1-618 KpnI/SnaBI Leu2)***	this study
YYW124	YYW120	*MATalpha hisΔ200 ura3-52 leu2-3,112* ***rpb1Δ187:: HIS3 +pWY043(pYIA CTD-fusion-Set2 KpnI/SnaBI Leu2)***	this study
YYW125	YYW121	*MATalpha hisΔ200 ura3-52 leu2-3,112* ***rpb1Δ187:: HIS3 ΔSET2::HPH+pWY044(pYIA CTD-fusion-Set2 1-618 KpnI/SnaBI Leu2)***	this study
YYW126	YYW121	*MATalpha hisΔ200 ura3-52 leu2-3,112* ***rpb1Δ187:: HIS3 ΔSET2::HPH+pWY043(pYIA CTD-fusion-Set2 KpnI/SnaBI Leu2)***	this study
YYW127	BY4742	*MATalpha his3Δ1 leu2Δ0 lys2Δ 0 ura3Δ0* ***Set2 1-261-C-TAP ::URA3***	this study
YYW129	YBL699	*MATa his3Δ1 leu2Δ0 met15Δ0 ura3Δ0* ***ΔRPH1::KAN ΔJHD1::Cre-LEU2 Set2 1-261-C-TAP::URA3***	this study
YYW132	YJW594	*MAT****a****, his3Δ1 leu2Δ0 met15Δ0 ura3Δ0* ***ΔRCO1::KANMX6 ΔSET2::URA3***	this study
YYW134	YYW122	*MATa ura3-52 his3Δ200 leu2Δ1 or Δ0 trp1Δ63 met15Δ0 lys2-128 ∂ gal10Δ56 rpb1Δ::CLONATMX* ***Rpb3-TAP::KlacTRP1+pWY045 (pYIA CTD-fusion-Set2 1-261 KpnI/SnaBI Leu2)***	this study

YYW136	YYW122	*MATa ura3-52 his3Δ200 leu2Δ1 or Δ0 trp1Δ63 met15Δ0 lys2-128 ∂ gal10Δ56 rpb1Δ::CLONATMX* ***Rpb3-TAP::KlacTRP1+pWY045 (pYIA CTD-fusion-Set2 1-618 KpnI/SnaBI Leu2)***	this study
YYW138	YYW122	*MATa ura3-52 his3Δ200 leu2Δ1 or Δ0 trp1Δ63 met15Δ0 lys2-128 ∂ gal10Δ56 rpb1Δ::CLONATMX* ***Rpb3-TAP::KlacTRP1+pWY045 (pYIA CTD-fusion-Set2 KpnI/SnaBI Leu2)***	this study
YYW162	W303	*MATa ade2 can1 his3 leu2 lys2 met15 trp1 ura3* ***spt16-11***	D. Stillman
YYW163	W303	*MATa ade2 can1 his3 leu2 ura3* ***spt16-11 ΔSET2::KAN***	D. Stillman
YYW165	YYW163	*MATa ade2 can1 his3 leu2 ura3* ***spt16-11 ΔSET2::KAN ΔRCO1::cre-HIS(c.kl)***	this study
YYW800	YYW010	*MATalpha his3Δ1 leu2Δ0 lys2Δ 0 ura3Δ0* ***ΔSET2::KAN+pWY129 (pRS416-ADH1pro-FLAG-Set2 ΔA(Δ262-302))***	this study
YYW801	YYW010	*MATalpha his3Δ1 leu2Δ0 lys2Δ 0 ura3Δ0* ***ΔSET2::KAN+pWY137 (pRS416-ADH1pro-FLAG-Set2 ΔB(Δ303-338))***	this study
YYW802	YYW010	*MATalpha his3Δ1 leu2Δ0 lys2Δ 0 ura3Δ0* ***ΔSET2::KAN+pWY136 (pRS416-ADH1pro-FLAG-Set2 ΔC(Δ339-386))***	this study
YYW803	YYW010	*MATalpha his3Δ1 leu2Δ0 lys2Δ 0 ura3Δ0* ***ΔSET2::KAN+pWY138 (pRS416-ADH1pro-FLAG-Set2 ΔD(Δ387-415))***	this study
YYW804	YYW010	*MATalpha his3Δ1 leu2Δ0 lys2Δ 0 ura3Δ0* ***ΔSET2::KAN+pWY151 (pRS416-ADH1pro-FLAG-Set2 ΔE(Δ416-476))***	this study
YYW805	YYW010	*MATalpha his3Δ1 leu2Δ0 lys2Δ 0 ura3Δ0* ***ΔSET2::KAN+pWY152 (pRS416-ADH1pro-FLAG-Set2 ΔA-E(Δ216-476))***	this study
YYW806	YYW010	*MATalpha his3Δ1 leu2Δ0 lys2Δ 0 ura3Δ0* ***ΔSET2::KAN+pWY087 (pRS416-ADH1pro-FLAG-Set2 SET-G5-SRI-TEV(Δ262-618))***	this study
YYW807	YYW010	*MATalpha his3Δ1 leu2Δ0 lys2Δ 0 ura3Δ0* ***ΔSET2::KAN+pWY088 (pRS416-ADH1pro-FLAG-Set2 FL)***	this study
YYW808	YYW010	*MATalpha his3Δ1 leu2Δ0 lys2Δ 0 ura3Δ0* ***ΔSET2::KAN+pWY084 (pRS416-ADH1pro-APDT2-FLAG-CYC1ter)***	this study
YYW809	YYW162	*MATa ade2 can1 his3 leu2 lys2 met15 trp1 ura3* ***spt16-11+pWY084 (pRS416-ADH1pro-APDT2-FLAG-CYC1ter)***	this study
YYW810	YYW163	*MATa ade2 can1 his3 leu2 ura3* ***spt16-11 ΔSET2::KAN+pWY084 (pRS416-ADH1pro-APDT2-FLAG-CYC1ter)***	this study
YYW811	YYW163	*MATa ade2 can1 his3 leu2 ura3* ***spt16-11 ΔSET2::KAN+pWY088 (pRS416-ADH1pro-FLAG-Set2 FL)***	this study
YYW812	YYW163	*MATa ade2 can1 his3 leu2 ura3* ***spt16-11 ΔSET2::KAN+pWY129 (pRS416-ADH1pro-FLAG-Set2 ΔA(Δ262-302))***	this study
YYW813	YYW163	*MATa ade2 can1 his3 leu2 ura3* ***spt16-11 ΔSET2::KAN+pWY137 (pRS416-ADH1pro-FLAG-Set2 ΔB(Δ303-338))***	this study
YYW814	YYW163	*MATa ade2 can1 his3 leu2 ura3* ***spt16-11 ΔSET2::KAN+pWY136 (pRS416-ADH1pro-FLAG-Set2 ΔC(Δ339-386))***	this study
YYW815	YYW163	*MATa ade2 can1 his3 leu2 ura3* ***spt16-11 ΔSET2::KAN+pWY138 (pRS416-ADH1pro-FLAG-Set2 ΔD(Δ387-415))***	this study
YYW816	YYW163	*MATa ade2 can1 his3 leu2 ura3* ***spt16-11 ΔSET2::KAN+pWY151 (pRS416-ADH1pro-FLAG-Set2 ΔE(Δ416-476))***	this study
YYW817	YYW163	*MATa ade2 can1 his3 leu2 ura3* ***spt16-11 ΔSET2::KAN +pWY182(pRS416-ADH1pro-FLAG-Set2 ΔD-C201A)***	this study
YYW818	YYW163	*MATa ade2 can1 his3 leu2 ura3* ***spt16-11 ΔSET2::KAN +pWY184(pRS416-ADH1pro-FLAG-Set2 ΔB-C201A)***	this study
YYW819	YYW165	*MATa ade2 can1 his3 leu2 ura3* ***spt16-11 ΔRCO1::cre-HIS(c.kl) ΔSET2:KAN+pWY129(pRS416-ADH1pro-FLAG-Set2 ΔA(Δ262-302))***	this study
YYW820	YYW165	*MATa ade2 can1 his3 leu2 ura3* ***spt16-11 ΔRCO1::cre-HIS(c.kl) ΔSET2:KAN+pWY137(pRS416-ADH1pro-FLAG-Set2 ΔB(Δ303-338))***	this study
YYW821	YYW165	*MATa ade2 can1 his3 leu2 ura3* ***spt16-11 ΔRCO1::cre-HIS(c.kl) ΔSET2:KAN+pWY136(pRS416-ADH1pro-FLAG-Set2 ΔC(Δ339-386))***	this study

YYW822	YYW165	*MATa ade2 can1 his3 leu2 ura3* ***spt16-11 ΔRCO1::cre-HIS(c.kl) ΔSET2:KAN+pWY138(pRS416-ADH1pro-FLAG-Set2 ΔD(Δ387-415))***	this study
YYW823	YYW165	*MATa ade2 can1 his3 leu2 ura3* ***spt16-11 ΔRCO1::cre-HIS(c.kl) ΔSET2:KAN+pWY151(pRS416-ADH1pro-FLAG-Set2 ΔE(Δ416-476))***	this study
YYW824	YYW010	*MATalpha his3Δ1 leu2Δ0 lys2Δ 0 ura3Δ0* ***ΔSET2::KAN+pWY131(pRS415-Set2 Pro/Ter-HA-Set2)***	this study
YYW825	YYW010	*MATalpha his3Δ1 leu2Δ0 lys2Δ 0 ura3Δ0* ***ΔSET2::KAN+pWY126(pRS415-Set2 Pro/Ter-HA-Set2 ΔA(Δ206-303))***	this study
YYW826	YYW010	*MATalpha his3Δ1 leu2Δ0 lys2Δ 0 ura3Δ0* ***ΔSET2::KAN+pWY127(pRS415-Set2 Pro/Ter-HA-Set2 ΔA-B(Δ206-338))***	this study
YYW827	YYW010	*MATalpha his3Δ1 leu2Δ0 lys2Δ 0 ura3Δ0* ***ΔSET2::KAN+pWY215(pRS415-Set2 Pro/Ter-HA-Set2 ΔD (Δ387-415))***	this study
YYW828	YYW010	*MATalpha his3Δ1 leu2Δ0 lys2Δ 0 ura3Δ0* ***ΔSET2::KAN+pWY216(pRS415-Set2 Pro/Ter-HA-Set2 ΔB(Δ303-338))***	this study
YYW829	YYW010	*MATalpha his3Δ1 leu2Δ0 lys2Δ 0 ura3Δ0* ***ΔSET2::KAN+pWY217(pRS415-Set2 Pro/Ter-HA-Set2 ΔC(Δ339-386))***	this study
YYW830	YYW010	*MATalpha his3Δ1 leu2Δ0 lys2Δ 0 ura3Δ0* ***ΔSET2::KAN+pWY218(pRS415-Set2 Pro/Ter-HA-Set2 ΔE(Δ416-476))***	this study
YYW831	YYW010	*MATalpha his3Δ1 leu2Δ0 lys2Δ 0 ura3Δ0* ***ΔSET2::KAN+pWY069(pRS415-pGAL1-HA-Set2)***	this study
YYW832	YYW010	*MATalpha his3Δ1 leu2Δ0 lys2Δ 0 ura3Δ0* ***ΔSET2::KAN+pWY052(pRS415)***	this study
YYW833	YYW010	*MATalpha his3Δ1 leu2Δ0 lys2Δ 0 ura3Δ0* ***ΔSET2::KAN+pWY091(pRS415-ADH1pro-ADPT2-FLAG-Set2 FL-CYC1ter)***	this study
YYW834	YCR377	*MATa his3Δ1 leu2Δ0 lys2Δ 0 ura3Δ0* ***STE11 1840-HIS3 ΔSET2::HPH +pWY084(pRS416-ADH1pro-ADPT2-FLAG-CYC1ter)***	this study
YYW835	YCR377	*MATa his3Δ1 leu2Δ0 lys2Δ 0 ura3Δ0* ***STE11 1840-HIS3 ΔSET2::HPH +pWY088(pRS416-ADH1pro-FLAG-Set2 FL)***	this study
YYW836	YCR377	*MATa his3Δ1 leu2Δ0 lys2Δ 0 ura3Δ0* ***STE11 1840-HIS3 ΔSET2::HPH +pWY129(pRS416-ADH1pro-FLAG-Set2 ΔA(Δ262-302))***	this study
YYW837	YCR377	*MATa his3Δ1 leu2Δ0 lys2Δ 0 ura3Δ0* ***STE11 1840-HIS3 ΔSET2::HPH +pWY137(pRS416-ADH1pro-FLAG-Set2 ΔB(Δ303-338))***	this study
YYW838	YCR377	*MATa his3Δ1 leu2Δ0 lys2Δ 0 ura3Δ0* ***STE11 1840-HIS3 ΔSET2::HPH +pWY136(pRS416-ADH1pro-FLAG-Set2 ΔC(Δ339-386))***	this study
YYW839	YCR377	*MATa his3Δ1 leu2Δ0 lys2Δ 0 ura3Δ0* ***STE11 1840-HIS3 ΔSET2::HPH +pWY138(pRS416-ADH1pro-FLAG-Set2 ΔD(Δ387-415))***	this study
YYW840	YCR377	*MATa his3Δ1 leu2Δ0 lys2Δ 0 ura3Δ0* ***STE11 1840-HIS3 ΔSET2::HPH +pWY151(pRS416-ADH1pro-FLAG-Set2 ΔE(Δ416-476))***	this study
YYW841	YCR377	*MATa his3Δ1 leu2Δ0 lys2Δ 0 ura3Δ0* ***STE11 1840-HIS3 ΔSET2::HPH +pWY087(pRS416-ADH1pro-FLAG-Set2 SET-G5-SRI-TEV(Δ262-618))***	this study
YYW842	YYW132	*MATahis3Δ1 leu2Δ0 met15Δ0 ura3Δ0* ***ΔRCO1::KAN ΔSET2::URA3+pWY131(pRS415-Set2 Pro/Ter-HA-Set2)***	this study
YYW843	YYW132	*MATahis3Δ1 leu2Δ0 met15Δ0 ura3Δ0* ***ΔRCO1::KAN ΔSET2::URA3+pWY126(pRS415-Set2 Pro/Ter-HA-Set2 ΔA(Δ206-303))***	this study
YYW844	YYW132	*MATahis3Δ1 leu2Δ0 met15Δ0 ura3Δ0* ***ΔRCO1::KAN ΔSET2::URA3+pWY127(pRS415-Set2 Pro/Ter-HA-Set2 ΔA-B(Δ206-338))***	this study

Table S3. List of plasmids.

Primer	Sequence	Notes
P747	TTACGGTCCATTTGAGGAATCACTG	STE11+1641F
P1160	CGACTCGAGcgttcaagaatctctctctc	SET2-Pro5XhoI
P1162	TCTGGATCCttaaccagacatgcatatttggg	SET2-Term5BamHI
P1163	CTAGCGGCCGCTAGTCCTATGTACAAGCACA	SET2-Term3NotI
P1255	ttcttggtcaaatggctcca	Rump-F
P1273	tctgagctcGTTACTTGATTCCCTATCCCTACCA	Fluff-R-SacI
P1397	TGTGATATCGGGAGTGCAAGGTCG	STE11 A up
P1398	ccggttaccgaggctgtatcaaagg	STE11 A down
P1399	GGAGGAAATAGGATGCACTCAATACCTGGATAG	STE11 B up
P1400	catcgatagcgtagcagtggatagagagc	STE11 B down
P1401	GCCATTCAGCAAGGCTAAGCATTGC	STE11 C up
P1402	ctggccttttatgggcaagcttcg	STE11 C down
P1403	GATTGGGGTTGCGTATTTGCATAAG	STE11 D up
P1404	gatgctgcagcaattcaagggc	STE11 D down
P1430	AGACTCGAGatgtcgaagaaccaaagtgtg	SET2-5XhoI+ATG
P1431	CTCGCGGCCGCTTATGATGATGTTGAAGGTG	SET2-3STOP-NotI
P1470	agaCTCGAGCCCGGGTCTGGAATTTTCATTTTCATTATGCT	RPB1-fusion-UpR
P1471	agactcgagtacccatacgatgttcctgactatgcgtgatatagtatatcatccttacgt	RPB1-fusion-DnF
P1472	AGACTCGAGACCACCGAGAAACCC	SET2-261R-NoSTOP-XhoI
P1561	agactcgagTTTCAACCGCTTAGCCTC	CTD-SET2-618RXhoI
P1562	agactcgagTGATGATGTTGAAGGTGGAGGA	CTD-SET2RXhoI
P1574	gcTAATACGACTCACTATAGGGAGAAATTATGTGTGCATC CAGCCA	T7-STE11-3R
P1612	acaGGATCCCTGGAAGTACAGGTTTTCACCACCGAGAAAC CCAATACA	SET2-261R-TEV-BamH1
P1613	acaGGATCCaagactcaaacagatgcggca	SET2-261F-BamH1
P1642	TAAGGATCCTTACGCATAGTCAGGAACATCGTATGGGTAC CCGGGGATCACGTAAAACAACAACCA	SET2-Pro3Sma-HA-TAA BamHI
P1695	[Phos]CTAGAatggattacaaggatgacgacgataagCTCGAGCCCGGGGC GGCCGC G	pADH1-Adpr2 X-Flag-XSNS-F adaptor
P1696	[Phos]TCGACGCGGCCGCCCCGGGCTCGAGcttatcgtcgtcatccttg taatccatT	pADH1-Adpr2 X-Flag-XSNS-F adaptor
P1728	acaAGATCTACCACCGAGAAACCCAATACA	SET2-261aa-R-BglII
P1733	acaCTCGAGGGATCCaagactcaaacagatgcggca	SET2-262-F-Xho1-Bam1
P1740	GTTTATAGCGGGCATTATGCGTAGATCAG	Z-4193F
P1741	GTTCCTCTAGAATTTTTCCACTCGCACATTC	Z-4477R

References

1. Govind, C.K., Qiu, H., Ginsburg, D.S., Ruan, C., Hofmeyer, K., Hu, C., Swaminathan, V., Workman, J.L., Li, B. and Hinnebusch, A.G. (2010) Phosphorylated Pol II CTD recruits multiple HDACs, including Rpd3C(S), for methylation-dependent deacetylation of ORF nucleosomes. *Mol Cell*, **39**, 234-246.
2. Li, B., Howe, L., Anderson, S., Yates, J.R., 3rd and Workman, J.L. (2003) The Set2 histone methyltransferase functions through the phosphorylated carboxyl-terminal domain of RNA polymerase II. *J Biol Chem*, **278**, 8897-8903.
3. Li, B., Gogol, M., Carey, M., Lee, D., Seidel, C. and Workman, J.L. (2007) Combined action of PHD and chromo domains directs the Rpd3S HDAC to transcribed chromatin. *Science*, **316**, 1050-1054.
4. Yun, M., Ruan, C., Huh, J.W. and Li, B. (2012) Reconstitution of modified chromatin templates for in vitro functional assays. *Methods Mol Biol*, **833**, 237-253.
5. Li, B. and Reese, J.C. (2001) Ssn6-Tup1 regulates RNR3 by positioning nucleosomes and affecting the chromatin structure at the upstream repression sequence. *J Biol Chem*, **276**, 33788-33797.
